# Simple sacrificial-layer-free microfabrication processes for air-cavity Fresnel acoustic lenses (ACFALs) with improved focusing performance

**DOI:** 10.1038/s41378-022-00407-w

**Published:** 2022-07-05

**Authors:** Yongkui Tang, Eun Sok Kim

**Affiliations:** Department of Electrical and Computer Engineering, University of Southern California, Los Angeles, CA 90089-0271 USA

**Keywords:** Electrical and electronic engineering, Electronic devices

## Abstract

Focused ultrasound (FUS) is a powerful tool widely used in biomedical therapy and imaging as well as in sensors and actuators. Conventional focusing techniques based on curved surfaces, metamaterial structures, and multielement phased arrays either present difficulties in massively parallel manufacturing with high precision or require complex drive electronics to operate. These difficulties have been addressed by microfabricated self-focusing acoustic transducers (SFATs) with Parylene air-cavity Fresnel acoustic lenses (ACFALs), which require a time-demanding step in removing the sacrificial layer. This paper presents three new and improved types of ACFALs based on polydimethylsiloxane (PDMS), an SU-8/PDMS bilayer, and SU-8, which are manufactured through simple sacrificial-layer-free microfabrication processes that are two to four times faster than that for the Parylene ACFALs. Moreover, by studying the effect of the lens thickness on the acoustic transmittance through the lens, the performance of the transducers has been optimized with improved thickness control techniques developed for PDMS and SU-8. As a result, the measured power transfer efficiency (PTE) and peak output acoustic pressure are up to 2.0 and 1.8 times higher than those of the Parylene ACFALs, respectively. The simple microfabrication techniques described in this paper are useful for manufacturing not only high-performance ACFALs but also other miniaturized devices with hollow or suspended structures for microfluidic and optical applications.

## Introduction

Focused ultrasound (FUS) has been used in a wide range of applications, including tumor ablation^1^, transcranial neuromodulation^2^, drug delivery^3^, contactless trapping^4^, acoustic droplet ejection^5^, wireless power transfer^6^, and nondestructive testing^7^. With acoustic energy focused on a small volume, FUS exhibits better performance than its unfocused counterpart in applications where high intensity or fine spatial resolution is desirable^8–10^.

To effectively focus ultrasound, acoustic waves generated from a vibrating sound source need to be designed to arrive at a focal point in phase. A straightforward way to achieve this is to create a curved transducer surface^11,12^ or attach a curved acoustic lens onto a flat transducer^5,13^. However, such surfaces are usually fabricated through macromachining techniques, including milling and heat pressing, whose limited precision may lead to fabrication defects, including surface roughness and curvature error. Alternatively, acoustic waves could be focused by programming the time delay of the driving signal applied on each transducer element in a phased array^14,15^. Through this approach, the focal position and acoustic beam direction can be precisely and dynamically controlled. However, phased array systems are typically bulky and expensive, with complicated drive electronics and many electrical connections to the transducer elements. A third way to realize acoustic focusing is to construct acoustic lenses based on metamaterials that can exhibit extraordinary properties such as wide bandwidth^16^ or high transmission^17^. However, due to their complex structures, the fabrication of these lenses is very challenging.

A simple and effective method to focus ultrasound is to utilize a thin and planar Fresnel acoustic zone plate^18^ that has a small footprint and can be microfabricated with high precision in a massively parallel manner. A simple implementation of this design is to pattern the top and bottom electrodes by sandwiching a piezoelectric substrate^19,20^ into Fresnel ring patterns through wet etching so that only acoustic waves contributing to constructive interference will be generated in the electrode-ring regions. However, this type of transducer suffers from fringing electrical fields, which produce non-thickness vibration modes^21^, heat generation due to the large series resistance of the electrodes, and tight front-to-back alignment tolerance during fabrication. A different approach is to create dual-layer^22^ or multilayer^23^ Fresnel acoustic lenses microfabricated through wet etching or reactive ion etching (RIE) and bond them to piezoelectric substrates. However, these lenses require critical layer thickness control to ensure good focusing and are time-consuming to fabricate since multiple layers are involved.

On the other hand, our previously demonstrated self-focusing acoustic transducers (SFATs) with Parylene air-cavity Fresnel acoustic lenses (ACFALs)^24,25^ are easy to microfabricate without tight requirements on thickness control or front-to-back alignment. As a result, they have been successfully applied in applications such as cancer treatment^26^, droplet ejection^25^, and underwater propulsion^27^. However, the fabrication of these Fresnel lenses is not time-efficient due to a sacrificial-layer release step required to create the air cavities, which may take several days to finish. Additionally, to open the release holes on the Parylene film, an RIE process is needed, which is time-consuming and requires tight alignment tolerance between the release holes and the rings. Another limitation of using Parylene as the lens material is the limited acoustic matching performance, since a lens thickness of a quarter wavelength is usually implemented to achieve the highest acoustic energy transmission^28^, which becomes unrealistically thick for the dimer-based vapor deposition of Parylene when the device is designed for frequencies lower than several MHz.

To make the microfabrication process of SFAT simpler and more time-efficient, we previously developed a new type of SFAT based on an ACFAL made of polydimethylsiloxane (PDMS), which was fabricated through soft lithography and ultraviolet (UV) epoxy bonding^21^. However, the bonding strength between the UV-curable epoxy and PDMS is too weak to survive the low-pressure Parylene deposition for electrical encapsulation, requiring additional steps. Additionally, the spin-coated PDMS exhibits nonuniform thickness across the lens due to the formation of edge beads and poor thickness repeatability between devices due to the viscosity of PDMS increasing over time after mixing.

This paper describes optimized sacrificial-layer-free microfabrication processes for the PDMS ACFAL and newly developed ACFALs based on an SU-8/PDMS bilayer and SU-8. All three types of ACFALs can be microfabricated two to four times faster than the Parylene ACFAL and offer better focusing performance, including higher output acoustic pressure and higher power transfer efficiency. The design and fabrication of newly developed SFATs are presented, and the measurement results of these devices are compared with each other, as well as against previously demonstrated SFATs based on Parylene ACFAL^25^ and patterned electrode rings^19^.

## Results

### Focusing principle of the air-cavity Fresnel acoustic lens (ACFAL)

**Fig. 1:**
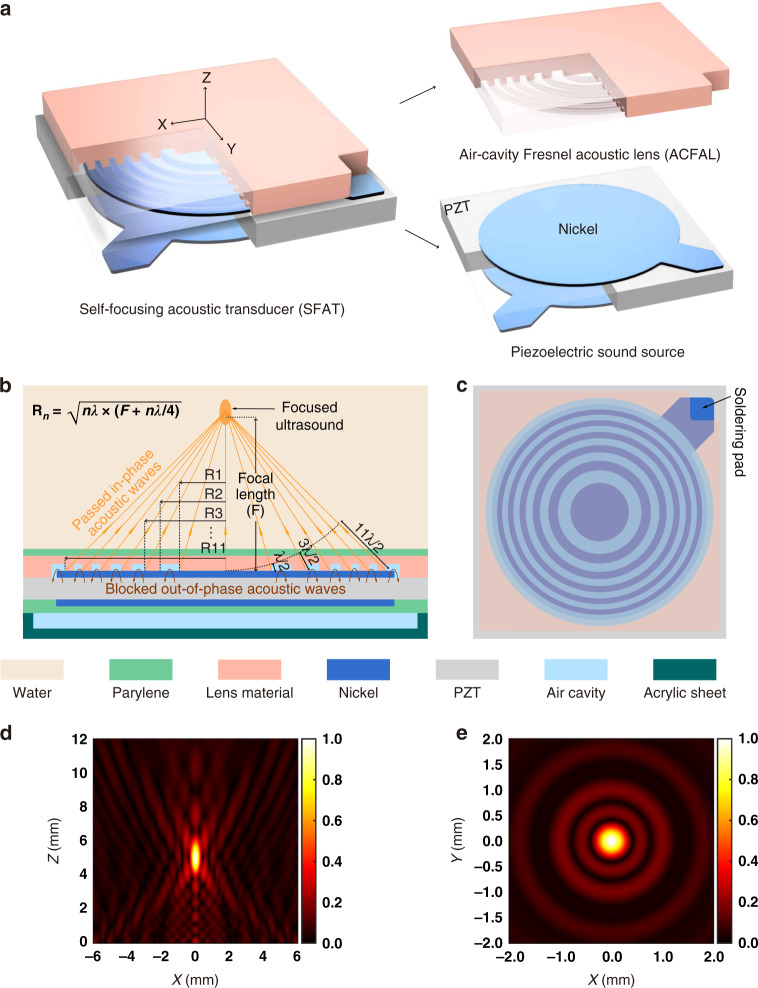
Schematic diagrams and acoustic pressure distributions of an SFAT with an air-cavity Fresnel acoustic lens (ACFAL). **a** Perspective schematic diagrams showing an SFAT consisting of a PZT sound source with patterned electrodes and an ACFAL with annular-ring air cavities. Quadrant portions of the ACFAL and PZT are made translucent to better illustrate the structure of the transducer. **b** Cross-sectional diagram of an SFAT, showing how the ACFAL focuses ultrasound by blocking destructively interfering acoustic waves. **c** Top-view diagram of the transducer showing the relative positions of the top electrode (and soldering pad), air-cavity rings, and non-air-cavity regions. **d**, **e** FEM-simulated normalized acoustic pressure in water at 2.32 MHz from an ideal ACFAL with six non-air-cavity regions designed for a 5-mm focal length (**d**) on the central vertical plane and **e** the lateral focal plane at *Z* = 5 mm, with the same color bar scale but different dimension scales.

A typical SFAT consists of two parts (Fig. 1a–c): a piezoelectric ultrasonic sound source and an air-cavity Fresnel acoustic lens (ACFAL) for focusing. For the ACFAL-based SFATs demonstrated in this paper, the sound source is a piezoelectric 1-mm-thick lead zirconate titanate (PZT) substrate sandwiched by top and bottom circular electrodes overlapping with each other in the transducer center. To provide electrical connections, the top and bottom circular nickel electrodes are extended into two nonoverlapping soldering pads located at different corners of the transducers, where electrical wires are soldered (Fig. 1c). When a sinusoidal voltage at the fundamental thickness-mode anti-resonant frequency of the PZT substrate (~2.32 MHz) is applied to the electrodes, the PZT substrate vibrates in the thickness direction, generating ultrasound waves, which are focused through the microfabricated ACFAL on the top electrode (Fig. 1a, b). The devices are chosen to work at the fundamental anti-resonant frequency of the PZT substrate instead of the resonant frequency so that the energy conversion efficiency (mechanical output power over electrical input power) of the PZT is maximized due to significantly lower mechanical and electrical losses^29,30^, which are desirable for applications where high power or high efficiency is needed, such as tumor ablation and wireless power transfer.

Each type of ACFAL demonstrated in this work incorporates six annular-ring air cavities alternating with six non-air-cavity regions (one center circle and five rings), with the lens material uniformly covering the top electrode (Fig. 1a, c). To compare the performance of different types of ACFALs, all transducers described in this paper are based on the same substrate material, electrode shape (all circular except the ones based on patterned electrode rings), and ring pattern of the lens. The radii of the ring boundaries are designed to form Fresnel half-wavelength bands (FHWB)^22^ for a 5-mm focal length in water at 2.32 MHz, so that the path–length difference between two adjacent ring boundaries to the designed focal point equals a half-wavelength in water (Fig. 1b). As a result, the acoustic waves coming from non-air-cavity regions (including the center circle and the outside rings) propagate through the lens and arrive at the focal point partially in phase (with phase difference <180°) to interfere constructively and generate FUS. The out-of-phase waves generated in the air-cavity–ring regions (that would have contributed to destructive interference at the focal point), on the other hand, are almost completely blocked by the air cavities due to the large mismatch between the acoustic impedances of air (0.4 kRayl)^31^ and the lens material (over 1 MRayl; see Table S1).

For visualization of the generated FUS, the normalized acoustic pressure distribution is simulated with the finite element method (Supplementary Method S1). In the simulation, for generality, the acoustic waves passing through the lens are modeled as ring-shaped sound sources, while the thickness and material of the lens are not considered. From the simulation results, it is clear that over the central vertical plane (Fig. 1d), as expected, a focal zone with high acoustic pressure is located 5 mm above the transducer center with a 1298.3 μm depth of focus (DoF). On the focal plane at *Z* = 5 mm, the focal diameter is simulated to be 363.2 μm (Fig. 1e).

To prevent acoustic energy from leaking from the back (or bottom) side of the transducer and thus maximizing the acoustic energy emitted from the top toward the focal point, a large air cavity is created on the back side, covering the whole electrode area by attaching laser-machined acrylic sheets with waterproof superglue (Fig. 1b and Fig. S1; Supplementary Method S2).

### Overview of the newly developed ACFALs

**Fig. 2:**
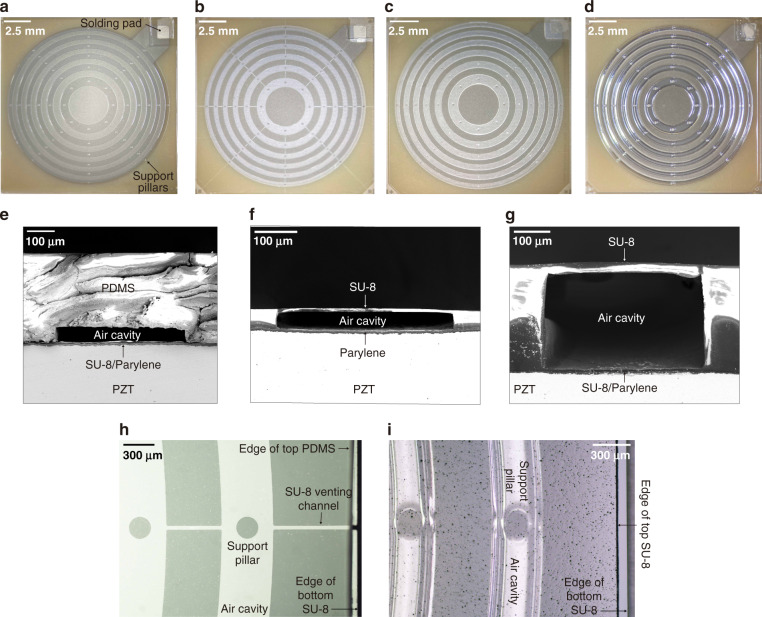
Photos of SFATs based on newly developed ACFALs. **a**–**d** Top-view photos of SFATs with **a** a PDMS ACFAL, **b** an SU-8/PDMS ACFAL, **c** a 45-μm-thick SU-8 ACFAL, and **d** a 283.5-μm-thick SU-8 ACFAL, all before electric wires are soldered. **e**–**g** Cross-sectional scanning electron microscope (SEM) photos of SFATs with **e** a PDMS ACFAL, **f** a 45-μm-thick SU-8 ACFAL, and **g** a 283.5-μm-thick SU-8 ACFAL. **h**–**i** Top-view microscope photos showing parts of **h** an SU-8/PDMS ACFAL and **i** a 283.5-μm-thick SU-8 ACFAL.

Using newly developed microfabrication processes, we successfully fabricated three types of SFATs with ACFALs based on PDMS (Fig. 2a, e), an SU-8/PDMS bilayer (Fig. 2b, h), and SU-8 (Fig. 2c, d, f, g, and i). These materials are commonly used in microfabrication and have good mechanical as well as acoustic properties, making them good structural materials for microfabricated acoustic lenses. For comparison, we also fabricated previously demonstrated SFATs based on Parylene ACFAL^25^ (Fig. S2) and patterned electrode rings^19^ (Fig. S3).

The detailed device information is listed in Table 1 with the microfabrication processes for different types of ACFALs summarized in Table 2, from which we can see that the fabrication time for all three new types of SFATs is two to four times faster than that for the Parylene-ACFAL-based SFATs, as fewer Parylene depositions and no RIE or sacrificial-layer release steps are involved.

**Table 1:** Summary of device information and measured focal parameters.

Device type	PDMS ACFAL (New)		SU-8/PDMS ACFAL (New)	SU-8 ACFAL (New)		Parylene ACFAL (Old)	Patterned electrode rings (Old)
Device annotation	P260	P350	S35/P245	S45	S284	PL	ER
Substrate	1-mm-thick PZT-5A						
Device area	16 × 16 mm^2^						
ACFAL layers (from PZT to water, with main layers highlighted in bold)	3.5 μm Parylene 3.5 μm SU-8 **260** **μm PDMS** 24 μm Parylene	3.5 μm Parylene 3.5 μm SU-8 **350** **μm PDMS** 24 μm Parylene	3.5 μm Parylene **35** **μm SU-8** **245** **μm PDMS** 24 μm Parylene	3.5 μm Parylene **45** **μm SU-8 (35** **μm bottom** + **10** **μm top)** 24 μm Parylene	3.5 μm Parylene **283.5** **μm SU-8 (253.5** **μm bottom** + **30** **μm top)** 24 μm Parylene	**27.5** **μm Parylene**	**27.5** **μm Parylene (no air cavities)**
Air-cavity height (μm)	50	50	35	35	253.5	2	NA
Measured anti-resonant frequency (MHz)	2.316	2.316	2.304	2.312	2.314	2.287	2.321
Measured focal length (mm)	5.04	5.14	5.05	4.98	4.70	4.98	4.97
Measured focal diameter (μm)	325.4	360.2	319.0	300.0	284.5	331.0	386.9
Measured depth of focus (μm)	1284.0	1190.4	1249.7	1362.2	1240.5	1350.0	1443.7

**Table 2:** Summary of the processes involved in the microfabrication of different types of SFATs.

Device type	PDMS ACFAL (new)	SU-8/PDMS ACFAL (new)	SU-8 ACFAL (new)	Parylene ACFAL (old)	Patterned electrode rings (old)
Photolithography (details and annotations shown in Table 3)	A + B (once per batch) G + H (one time only)	A + C	A + C + D (thin-SU-8) or A + B + E + F (thick SU-8)	A + sacrificial-layer patterning + release hole patterning (tight alignment tolerance)	A
Parylene deposition	1 (for electrical encapsulation, can be replaced by other sealants) + 1 (optional for adhesion promotion)	1 (for SU-8/PDMS bonding) + 1 (optional for adhesion promotion)	1 (for electrical encapsulation, can be replaced by other sealants) + 1 (optional for adhesion promotion)	2	1
RIE & Sacrificial-layer release	0	0	0	1 (time-consuming)	0
PDMS casting	0.25 (4 sheets/16 lenses per batch)	0.25 (4 sheets/16 lenses per batch)	0	0	0
Bonding	1	1	1 (laminator needed)	0	0
Fabrication time	~1 day	~1 day	~1 day (thin-SU-8) or ~2 days (thick SU-8 with thickness planarization)	~4 days (limited by sacrificial-layer release)	~0.5–1 day
Cleanroom equipment involved	Spinner, mask aligner, hot plate (for baking photoresist), plasma asher, fume hood			Spinner, mask aligner, oven (for baking photoresist), plasma asher, fume hood, RIE	Spinner, mask aligner, oven (for baking photoresist), fume hood
Non-cleanroom equipment involved	Parylene coater, vacuum desiccator, oven (for curing PDMS), stereomicroscope, dicing saw (for parallel fabrication)		Parylene coater, laminator, dicing saw	Parylene coater, dicing saw	Parylene coater, dicing saw
Measured peak output acoustic pressure (MPa)	0.68 (from P260)	0.74	1.10 (from S284)	0.61	0.28
Measured best power transfer efficiency (%)	15.37 (from P260)	15.20	30.13 (from S284)	15.13 (limited by Parylene thickness)	4.72 (limited by fringing electrical fields and non-thickness vibration modes)

**Fig. 3:**
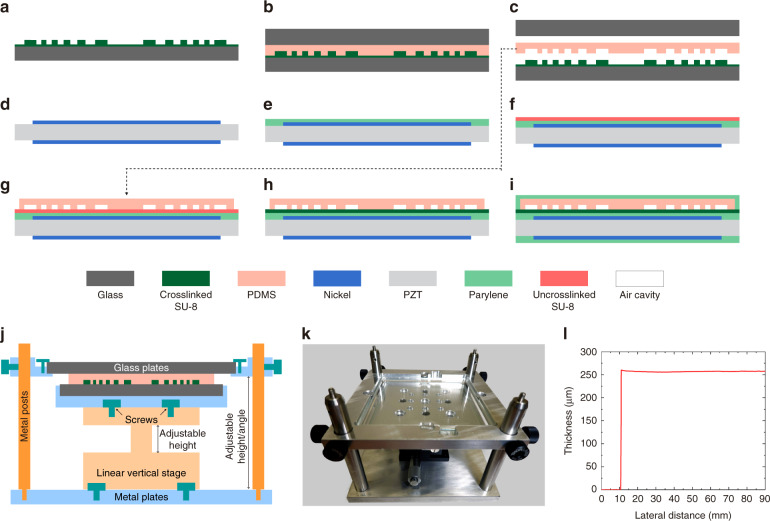
Microfabrication processes for SFATs with PDMS ACFALs. **a**–**i** Microfabrication processes for the transducer (not to scale). On a glass plate, **a** create an SU-8 adhesion layer and SU-8 mold; **b** replicate the PDMS membrane from the SU-8/glass mold, control the PDMS thickness with the aid of a clamping mechanism (Figs. 3j, k) and another blank glass plate; **c** detach the PDMS membrane from the glass plates. On PZT sheet, **d** pattern top/bottom electrodes; **e** deposit Parylene for improved adhesion to SU-8 (optional); **f** spin-coat thin-SU-8 and soft-bake; **g** trim the PDMS membrane to the desired size, align and attach it to the SU-8 layer on the PZT substrate; **h** soft-bake again to liquefy the SU-8 and initiate bonding between SU-8 and PDMS, followed by exposure and post-exposure bake to crosslink the SU-8; **i** solder electrical wires (not shown), then deposit Parylene for electrical encapsulation. (**j**–**k**) **j** Cross-sectional diagram and **k** photo of the clamping mechanism designed for precisely controlling the thickness of the PDMS membrane during curing. **l** Measured thickness profile of a fabricated PDMS membrane with good thickness uniformity across an 80-mm length.

For the PDMS-ACFAL-based SFATs, the air cavities are created by bonding a PDMS membrane (with grooves made through soft lithography) to a flat PZT surface. In this work, we improved the microfabrication processes (Fig. 3a–i) based on our previous work^21^. First, a thin layer of SU-8 photoresist replaces the previously used UV-curable epoxy as an adhesive to bond the PDMS lens to the PZT substrate, which not only offers good adhesion but also allows realignment if the initial alignment between the lens and the substrate is unsatisfactory. Previously, with UV-curable epoxy, even with an extra silane deposition step to improve bonding strength, the bonded layers would delaminate due to the pressure difference between the air cavities (at atmospheric pressure) and the low-pressure deposition chamber during the final Parylene deposition for electrical encapsulation. As a result, the electrical connections must be sealed by manually applying multiple coats of waterproof sealant. Now, with SU-8 as the adhesive, only a 1-min plasma treatment is necessary to ensure a strong bond that survives the low-pressure Parylene deposition. Second, we use SU-8 on a 4-inch square glass plate instead of a DRIE-etched 3-inch silicon wafer as the mold for PDMS casting, which is simpler, more time-efficient, and has higher throughput (since the effective area of the casted PDMS area is quadrupled). Moreover, we have developed an adjustable clamping mechanism that precisely controls the PDMS thickness by adjusting the vertical distance between the SU-8/glass mold and a blank glass plate sandwiching the PDMS during curing (Fig. 3j to l). From six PDMS castings using the clamping mechanism, the thickness variation across the same fabricated 260 µm thick PDMS membranes having a 90 mm side-length is only 31.6 ± 11.8 μm, causing the acoustic transmittance to drop from the maximum 16% only to ~15%, according to the calculation described in the next subsection. In smaller 16 × 16 mm^2^ lens areas, the average thickness variation is only 5.8 ± 4.2 μm, and in the best case, the thickness variation along an 80-mm-long scan length can be as small as 2 μm (Fig. 3l). The thickness variation is mainly caused by the imperfect parallelism between the two glass plates, as well as the bulging of the four-edge-clamped top glass plate due to thermal expansion. The thickness repeatability (error between the set thickness of 260 µm and the actual average thickness) is 30.5 ± 7.7 μm, which mainly comes from the error during the zeroing process of the mechanism and the precision of the height-controlling micrometer used in the linear movable stage. In comparison, if the PDMS thickness is controlled by spin-coating, the thickness variation across a short length of 35 mm can be over 70 μm (Fig. S4).

**Fig. 4:**
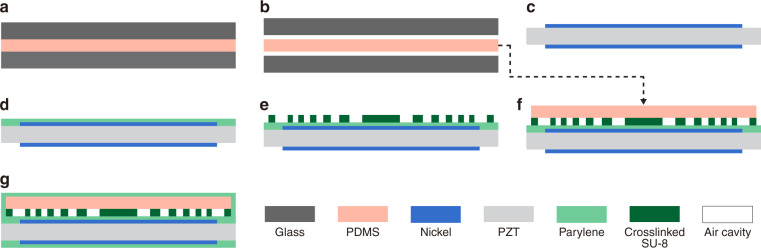
Microfabrication processes for SFATs with SU-8/PDMS ACFALs. **a**–**g** Microfabrication processes for the transducer (not to scale). On a glass plate, **a** create PDMS membrane from two blank glass plates using the thickness-controlling clamping mechanism, and **b** detach PDMS membrane from the glass plates. On PZT sheet, **c** pattern top/bottom electrodes; **d** deposit Parylene for improved adhesion to SU-8 (optional); **e** pattern bottom SU-8 layer through photolithography; **f** trim the PDMS membrane to the desired size, align and attach it to the patterned SU-8 bottom layer on the PZT substrate; **g** solder electrical wires (not shown), then deposit Parylene for sealing PDMS and SU-8 together.

To make the air cavities on the SU-8/PDMS ACFAL, we have developed a simple, heatless, adhesive-free technique to permanently attach a flat PDMS membrane (whose thickness is also controlled by the clamping mechanism) onto a patterned SU-8 bottom layer on the PZT through Parylene encapsulation (Fig. 4).

**Fig. 5:**
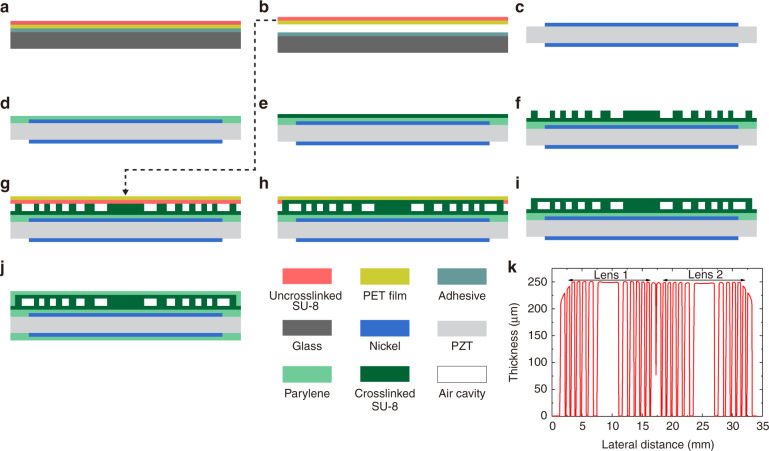
Microfabrication processes for SFAT-based SU-8 ACFALs. **a**–**j** Microfabrication processes for the transducer (not to scale). On a glass plate, **a** attach polyester (PET) film, spin-coat SU-8 top layer followed by long soft-bake, and **b** remove the PET film with SU-8 from the glass plate. On PZT sheet, **c** pattern top/bottom electrodes; **d** deposit Parylene for improved adhesion to SU-8 (optional); **e** create a thin-SU-8 adhesion layer (not necessary for SU-8 thinner than 50 μm); **f** pattern bottom SU-8 layer through photolithography; **g** bond top SU-8 (on PET film) to bottom patterned SU-8 layer on PZT with a laminator; **h** crosslink top SU-8 layer through exposure; **i** peel off PET film from SU-8, then remove uncrosslinked top SU-8 through development; **j** solder electrical wires (not shown), then deposit Parylene for electrical encapsulation. **k** Thickness profile of a 250-μm-thick patterned bottom SU-8 layer for two adjacent ACFALs after planarization, showing good thickness uniformity across most lens areas.

For the third type (i.e., SFATs with SU-8 ACFALs), the air cavities are created by bonding a thin and flat SU-8 top layer supported by a thin polyester (PET) film onto a thicker and patterned SU-8 bottom layer (created on the PZT substrate) with a laminator^32^ (Fig. 5a–j). To optimize the power transmission efficiency through the lens, the bottom SU-8 thickness can be precisely controlled through spin-coating followed by a planarization step^33^. As a result, we have been able to create an SU-8 bottom layer as thick as 250 μm with a small thickness variation of 6% across the whole lens (Fig. 5k), which is much smaller than the 57% thickness variation without the planarization step (Fig. S5).

### Thickness control for focusing performance optimization and measurement results

**Fig. 6:**
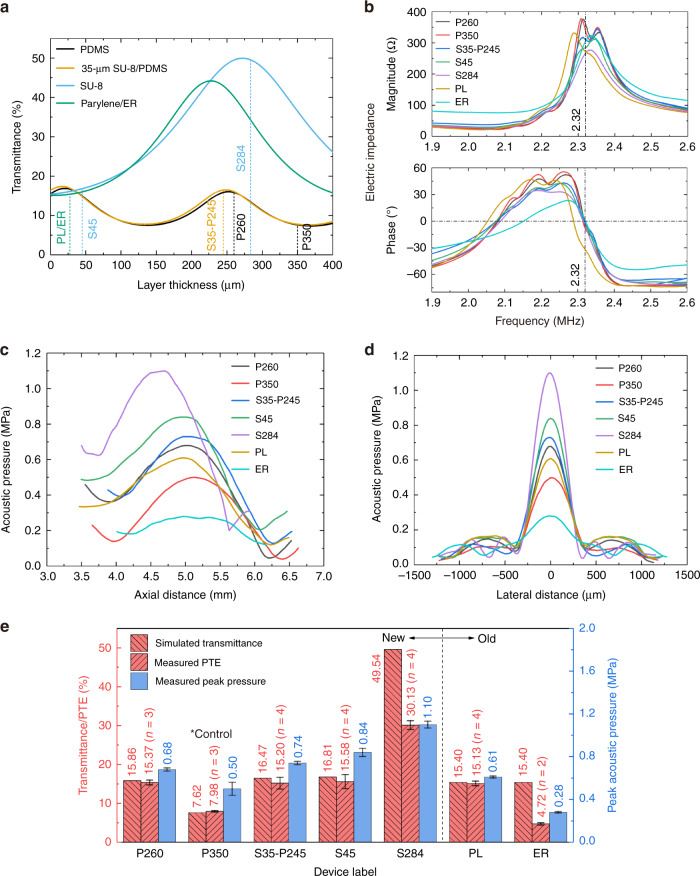
Thickness optimization simulation and measurement results. Thickness optimization simulation and measurement results. **a** Simulated acoustic transmittance versus main layer thickness for SFATs with ACFALs based on PDMS, 35-μm-thick SU-8/PDMS, SU-8, and Parylene (also for the electrode-ring SFATs); also showing the chosen layer thicknesses for the devices demonstrated in this paper along with their notations (with details shown in Table 1). **b** Measured impedance magnitude (upper graph) and phase (lower graph) of the fabricated transducers. **c**–**d** Measured acoustic pressure in water (**c**) along the central vertical axis and **d** along the central lateral axis on the focal plane of the fabricated devices when they are driven with six cycles of 40 V_pp_ sinusoidal voltage signals at their anti-resonant frequency. **e** Summary of the simulated acoustic transmittance (left *y*-axis), measured power transfer efficiency (left *y*-axis), and measured peak acoustic pressure at the focal point (right *y*-axis) of each type of transducer. The error bars represent the standard deviation of the measured values.

Another important function of ACFAL is to provide acoustic impedance matching between the PZT substrate and the transmission medium (water) whose acoustic impedances are very different, being 36.19 and 1.48 MRayl, respectively (Table S1). To simulate the acoustic transmittance through each type of ACFAL, a one-dimensional (1D) acoustic transmission line model^34,35^ is used due to its simplicity and effectiveness. For this task, although a multiphysics FEM simulation coupling the acoustics, electronics, and solid mechanics equations will ideally give more accurate results, the lack of published material properties in each physical domain limits its accuracy and thus has not been used. From the simulation results (Fig. 6a), we see that with Parylene, although the maximal achievable transmittance is as high as 44.2%, the maximum can only be achieved with a thickness of 227 μm, which is too thick for a typical Parylene deposition process based on pyrolysis and evaporation of the Parylene dimer. For demonstration, Parylene with a realistic thickness of 27.5 μm (which is already considered very thick) is chosen as the structure layer of the Parylene ACFAL (labeled “PL”) and the encapsulation layer for the SFAT with patterned electrode rings (labeled “ER”). For lenses made of PDMS, although the maximal achievable transmittance is lower than that of Parylene due to the lower acoustic impedance of PDMS (Table S1), theoretical transmittances of 16.9% and 16.0% could be achieved with thicknesses of 22 μm and 252 μm, respectively. Here, we pick the higher thickness for easy handling of the PDMS membrane. Additionally, when the thickness is ~252 μm, the transmittance is so insensitive to the thickness variation that it will only drop to 15.0% for a thickness variation of ±18 μm. In this paper, to demonstrate the effect of thickness optimization, we present two types of PDMS ACFAL with PDMS thicknesses of 260 μm (labeled “P260”) and 350 μm (labeled “P350”), which have theoretical transmittances of 15.86% (close to the maximal value) and 7.62% (close to the minimal value), respectively. For the SU-8/PDMS lens, if we fix a thickness of 35 μm for the bottom SU-8 layer (for ease of fabrication), a maximum transmittance of 17.4% and 16.5% can be achieved with PDMS thicknesses of 16.5 μm and 248.6 μm, respectively. For easy handling, we choose a thicker PDMS thickness of 245 μm (labeled “S35/P245”). Last, the SU-8-based lens provides the highest theoretical transmittance of 50.0% when the thickness is ~272 μm. For demonstration, we have made two types of SU-8 ACFAL with total SU-8 thicknesses of 283.5 μm (labeled “S284”, for 49.54% theoretical transmittance) and 45 μm (labeled “S45”, for 16.81% theoretical transmittance). The latter is faster to fabricate compared to the former, while still offering a theoretical transmittance higher than that of the PL.

The electrical impedances of the SFATs are measured with a vector network analyzer (Supplementary Method S3) to determine their anti-resonant frequencies (Fig. 6b and Table 1), at which the phase of the impedance is equal to zero and the impedance magnitude is near its maximum. The measured anti-resonant frequencies are all close to the designed 2.32 MHz (varying from 2.287 to 2.321 MHz with the deviation mainly due to the loading effect of the lens material, Table 1). Then, the acoustic pressure distribution of each SFAT driven with 40 V_pp_ pulsed sinusoidal voltage at the anti-resonant frequency is measured with a commercial hydrophone aligned to and scanned along the SFAT’s central vertical axis to determine the focal length, depth of focus (DoF), and peak acoustic pressure (Fig. 6c and Table 1), followed by a lateral scan along the focal plane to characterize the focal diameter (Fig. 6d and Table 1). The measured focal lengths are close to the designed 5 mm (from 4.70 to 5.14 mm, Table 1), with focal diameters and DoFs close to the simulated values of 363.2 μm (from 284.5 to 386.9 μm, Tables 1) and 1298.3 μm (from 1190.4 to 1443.7 μm, Table 1), respectively. The difference between these measured focal dimensions and simulated ones mainly comes from the thickness of the lenses, which is not considered in the FEM simulation (Fig. 1d, e).

As it is difficult to directly characterize the transmittance through the lenses, we characterize the power transfer efficiency (PTE), which is defined as the ratio of the output acoustic power over the applied real electric power. The measured peak acoustic pressure and PTE of each device are shown in Fig. 6e, from which we can see that the measured transfer efficiency is close to the estimated transmittance except for S284 and ER, and the higher the PTE is, the higher the peak acoustic pressure. Although the measured PTE is lower than the simulated value, S284 has the highest PTE of 30.13% and the highest peak acoustic pressure of 1.10 MPa among all the devices, which are 1.99 and 1.80 times higher than the measured values of the PL (15.13% and 0.61 MPa), respectively. In addition, P260, S35-P245, and S45, which are all manufactured through the new and faster microfabrication processes, have higher PTE (15.86%, 16.47%, and 16.81%, respectively) and peak acoustic pressure (0.68, 0.74, and 0.84 MPa, respectively) than those of the PL. Additionally, by comparing the measurement results of the unoptimized P350 (7.98% and 0.5 MPa) and the optimized P260 (15.86% and 0.68 MPa), we see that thickness optimization results in significant improvement in the focusing performance. Last, although the ER should theoretically have a similar performance compared to the PL, its actual performance is much worse (4.72% and 0.28 MPa), in agreement with previous experiments^22^. This is likely due to fringing electrical fields between electrode-ring pairs across the thickness direction and non-thickness vibration modes when the electrode-ring width is close to or smaller than the thickness of the PZT substrate.

## Discussion

This paper presents three new types of SFATs with ACFALs based on PDMS, SU-8/PDMS, and SU-8, which not only can be microfabricated two to four times faster than the previously demonstrated SFATs based on Parylene ACFALs made with a sacrificial (or spacer) layer but also have better PTE (up to 30.13%, which is two times higher) and higher peak output acoustic pressure (up to 1.10 MPa with 40 V_pp_ applied, which is 1.8 times higher), which make them better tools for FUS-related applications such as tumor treatment and transcranial neuromodulation. Although the SFATs based on patterned electrode rings are even easier to fabricate due to the lack of an air-cavity lens, they have poor focusing performance due to non-thickness vibration modes, which become substantial when the frequency is less than tens of MHz (at which the ring width is not much larger than the PZT thickness). To better evaluate the performance of our devices, we compared our devices with published high-efficiency acoustic transducers working at similar frequencies, with details summarized in Table S2. However, since most of these systems consist of a transmitting–receiving transducer pair, only the combined PTE is reported, which is the multiplication of the transmission PTE and the reception PTE, while for our devices, only the transmission PTE is characterized, as they are designed only for transmitting. Assuming the transmission and reception PTEs are equal in these systems (which may not be true, as the reception PTE can be over 90%^36^ for some systems), the estimated transmission PTE ranges from 1% to 71%, while the reported output pressure (normalized for 40 V_pp_ applied voltage) varies from 0.18 to 1.38 MPa, with the highest value achieved with a 64-element phased array. In comparison, our devices not only exhibit decent transmission efficiency and high output pressure but also have the advantages of being single-element and microfabricated.

The PTE values of the ACFAL-based SFATs estimated by a 1D acoustic transmission line model are close to the measured values except for S284, in which case the measured value is much lower, possibly due to (1) the tensile stress (stemming from the large thermal expansion coefficient^37^) of the thick SU-8 bending the PZT, (2) the surface roughness of the top SU-8 layer (the black “speckles” in Fig. 2i), shaped by the nonsmooth polyester (PET) film, and/or (3) the inaccuracy of the acoustic attenuation coefficient used in the calculation, as it is the value extracted from measurements at 1 GHz^38^. In the future, a more realistic multiphysics FEM simulation model of ACFAL in 2D or 3D, which considers the interaction between the lens material and the piezoelectric substrate, can be used to accurately predict the output pressure and PTE.

To further improve the performance of the devices, the substrate material can be changed to a piezoelectric composite, such as a 1–3 composite consisting of a lead magnesium niobate-lead titanate (PMN-PT) single crystal and epoxy, which has a lower loss, lower acoustic impedance, and higher electromechanical coupling coefficient^39,40^. Additionally, the acoustic impedance of the lens materials could be increased by adding titanium oxide (TiO_2_) nanoparticles^38,41^, which will bring the acoustic impedance of the lens material closer to the square root of the product of the acoustic impedances of PZT and water (7.32 MRayl) for better acoustic matching^31^. These two improvements can easily be incorporated into the processes described in this paper.

Apart from SFATs, the microfabrication techniques described in this paper can also be useful for fabricating other types of devices with hollow or suspended structures, including acoustic tweezers based on modified ACFALs^42^, microfluidic devices, optical waveguides, and 3D photonic crystals. Unlike similar processes for making hollow or suspended structures, including methods based on (1) the removal of a spacer (or sacrificial) layer through etching^25^ or high-temperature thermal depolymerization^43^, (2) additional additive fabrication steps such as electroplating^44^, (3) modified photolithography processes such as grayscale lithography^45^, moving-mask lithography^46^, holographic lithography^47^, or (4) lithography processes involving deposited metal mask^48^, image reversal photoresist^49^, and deep UV exposure sources that mismatch the absorption wavelength of the photoresist^50^, the approaches described in this paper are simple, time-efficient, and do not rely on complicated equipment, complex setup, or very high temperature (Tables 2 and 3). In fact, only the photolithography steps have to be carried out in a cleanroom, while other processes can be done in a regular wet lab. Moreover, the fabrication methods described in this paper are not mutually exclusive and can be flexibly combined according to available equipment and fabrication needs. For example, the Parylene encapsulation method for making SU-8/PDMS SFATs can also be used to fabricate PDMS SFATs. Alternatively, the lamination-based SU-8 bonding method can also be used for bonding PDMS to SU-8.

**Table 3:** Details of the photolithography processes described in this work.

Photolithography process	For electrode patterning on PZT	3.5-μm-thick SU-8 adhesion layer	35-μm-thick SU-8 bottom layer	10-μm-thick SU-8 top layer	250-μm-thick SU-8 bottom layer	30-μm-thick SU-8 top layer	2-μm-thick SU-8 adhesion layer on glass	50-μm-thick SU8 mold for PDMS casting
Label for reference	A	B	C	D	E	F	G	H
Cleaning or surface treatment	Rinse with acetone, methanol, IPA, and DI water; Blow-dry with N_2_.	30 s O_2_ plasma^a^	30 s O_2_ plasma^a^	Wipe with IPA; Blow-dry with N_2_.	30 s O_2_ plasma^a^	Wipe with IPA; Blow-dry with N_2_.	Piranha solution^b^ cleaning (20 min); Dehydration bake @ 175 °C (30 min) on a hot plate.	30 s O_2_ plasma^a^
Photoresist	AZ 5214 (Integrated Micro Materials)	SU-8 2005 (Kayaku Advanced Materials)	SU-8 2050 (Kayaku Advanced Materials)	SU-8 2005 (Kayaku Advanced Materials)	SU-8 2100 (Kayaku Advanced Materials)	SU-8 2050 (Kayaku Advanced Materials)	SU-8 2002 (Kayaku Advanced Materials)	SU-8 3050 (Kayaku Advanced Materials)
Spin-coating (spin speed, acceleration, duration of each step)	500 rpm, 500 rpm/s, 5 s; 1200 rpm, 600 rpm/s, 55 s.	500 rpm, 100 rpm/s, 10 s; 3000 rpm, 300 rpm/s, 60 s; 1,000 rpm, 500 rpm/s, 10 s.	500 rpm, 100 rpm/s, 10 s; 3000 rpm, 300 rpm/s, 60 s; 1,000 rpm, 500 rpm/s, 10 s.	On 75-μm-thick PET film: 500 rpm, 100 rpm/s, 10 s 600 rpm, 300 rpm/s, 30 s	500 rpm, 100 rpm/s, 10 s; 1450 rpm, 300 rpm/s, 30 s; Followed by thickness planarization.	On 38-μm-thick PET film: 500 rpm, 100 rpm/s, 10 s; 3500 rpm, 300 rpm/s, 60 s; 1,000 rpm, 500 rpm/s, 10 s.	500 rpm, 100 rpm/s, 10 s; 3000 rpm, 300 rpm/s, 30 s; 1000 rpm, 500 rpm/s, 10 s.	500 rpm, 100 rpm/s, 10 s; 3000 rpm, 300 rpm/s, 30 s; 1000 rpm, 500 rpm/s, 10 s.
Soft-bake (on a hot plate unless otherwise specified)	90 °C (5 min) in a convection oven	5 °C/min ramp rate RT → 95 °C (1 min) → RT	5 °C/min RT → 65 °C (1 min) → 95 °C (4 min) → RT	5 °C/min 65 °C → 90 °C (15 min) → RT	2 °C/min RT → 65 °C (5 min) → 95 °C (65 min) → RT	5 °C/min RT → 65 °C (5 min) → 90 °C (25 min) → RT	30 °C (30 min)	2 °C/min RT → 65 °C (70 min) → RT
SU-8/SU-8 bonding	NA	NA	1 min plasma treatment on bottom layer. Bonding in a laminator (Tamerica TCC6000) with speed level 3 @ 80 °C.		1 min plasma treatment on bottom layer. Bonding in a laminator with speed level 3 @ 80 °C.		NA	NA
Exposure dose (mJ/cm^2^)	140	150 (without photomask)	120	170	250	230	900 (without photomask)	800
Post-exposure bake (PEB) (on a hot plate)	NA	5 °C/min RT → 95 °C (hold 2 min) → RT	5 °C/min RT → 65 °C (1 min) → 95 °C (5 min) → RT	5 °C/min RT → 65 °C (1 min) → 90 °C (2 min) → RT	2 °C/min RT → 65 °C (5 min) → 95 °C (11 min) → RT	5 °C/min RT → 65 °C (2 min) → 90 °C (5 min) → RT	2 °C/min RT → 55 °C (60 min) → RT	2 °C/min RT → 55 °C (60 min) → RT
Development	70 s in AZ 400 K (1:4 diluted) (Integrated Micro Materials)	NA	6.5 min in PGMEA (Kayaku Advanced Materials)	2.5 min in PGMEA	22 min in PGMEA with stirring	5 min in PGMEA	NA	7 min in PGMEA
Hard bake (in a convection oven)	90 °C (15 min)	NA	NA	NA	NA	NA	10 °C/min RT → 150 °C (30 min) → RT	10 °C/min RT → 110 °C (30 min) → RT

*RT* room temperature, *PGMEA* propylene glycol monomethyl ether acetate.

^a^Plasma treatment condition: 35 W, 265 mTorr.

^b^Piranha solution: H_2_SO_4_ and 30% H_2_O_2_ with a volume ratio of 3:1.

## Materials and methods

### Microfabrication of the piezoelectric sound source

The fabrication of all the SFATs starts from a 1-mm-thick square PZT-5A substrate with predeposited 100-nm-thick nickel electrodes on both sides (Piezo.com). On one square PZT substrate with a side-length of 36.2 mm, four square SFATs with side lengths of 16.0 mm can be fabricated in parallel (Fig. S6) and separated by wafer dicing after fabrication. The top and bottom electrodes are patterned through photolithography (process A in Table 3) and wet etching at 30 °C (Nickel etchant TFG, Transene Company Inc.). The front-to-back alignment is achieved by aligning one corner of the PZT substrate to reference corners on the photomasks. For the SFATs based on electrode rings, the electrodes are patterned into Fresnel ring patterns connected by a rectangular electrode (Fig. S3), while circular electrodes (along with cross-shaped alignment markers) are patterned for the other ACFAL-based SFATs (Figs. 3d, 4c, 5c and Fig. S7).

As an optional step for SFATs having an SU-8 layer, the adhesion between the bottom SU-8 layer and PZT/nickel can be improved through deposition (with a PDS 2010 Parylene Coater, Specialty Coating Systems Inc.) of 3.5-μm-thick Parylene (DPX-D, Specialty Coating Systems Inc.) (Figs. 3e, 4d, and 5d). However, since the adhesion between SU-8 and PZT/nickel is already very good, this Parylene layer is not needed for most cases unless the SU-8 layer on the lens is very thick (for example, over 200 μm thick). After this, an ACFAL is fabricated on the PZT according to the steps described in the following subsections.

### Microfabrication of PDMS ACFAL

The microfabrication processes for SFATs with PDMS ACFAL are illustrated in Fig. 3a–i. First, an SU-8 mold is patterned on a 4-inch square glass plate (process H in Table 3) with a 2-μm-thick flat SU-8 as an adhesion-promotion layer (process G in Table 3) (Fig. 3a). The areas with SU-8 patterns will later form air cavities on the PDMS membrane after casting. To reduce the built-in stress in SU-8 to ensure its good adhesion to glass, a lower baking temperature with a slow ramp rate and longer baking time are used during the baking steps. The glass plate is large enough for four 36.2-mm-side-length patterns, which will generate sixteen 16 × 16 mm^2^ PDMS lenses per casting. To prevent hollow air cavities on the casted PDMS membrane from collapsing, we design the air gap height, which is equal to the SU-8 mold thickness, to be 50 μm (Fig. 2e). From our experiences with 260-μm-thick PDMS, the air-cavity height is recommended to be higher than 1/18 of the widest air-cavity ring width (790 μm in this case). For additional support, we also place eight 200-μm-diameter support pillars on each air-cavity ring (Fig. 2a). Although out-of-phase acoustic waves may pass through the support pillars, they occupy only 0.25% of the total active area, negligibly impacting the focusing. To prevent PDMS from permanently sticking to the glass after casting, the SU-8/glass mold is then hydrophobically silanized (SIH5841.0, Gelest Inc.). For the silane treatment, the mold is first treated with O_2_ plasma (35 W, 265 mTorr) for 1 min and then immediately put into a vacuum desiccator (Bel-Art Products Inc.) along with a glass slide with several drops (~0.2 mL) of silane on top, followed by pumping overnight.

Next, we cast PDMS onto the SU-8/glass mold to create the PDMS lenses (Fig. 3b). To precisely control the thickness of the PDMS (which is crucial to maximizing the PTE through the lens), a clamping mechanism was designed and machined (Fig. 3j, k). The clamping mechanism consists of three metal plates: one with a square opening, another with a recess (to hold glass plates), and the third as the platform base. The middle metal plate (with the recess), which holds the glass plate with an SU-8 mold, is fixed on a height-adjustable high-precision vertical linear movable stage (SEMZA-60, SF Technology Co., Ltd.), whereas the top metal plate holding a hydrophobically treated 4.5-inch square blank glass plate with screws is fixed on four metal posts (Thorlabs Inc.) with thumbscrews (Thorlabs Inc.) near its four corners. The linear stage and the four metal posts are all attached to the bottom metal plate. To create the PDMS lenses, base polymer and curing agent (Sylgard 184, Dow Inc.) are first mixed at a 10:1 weight ratio for 5 min and degassed for 50 min in a vacuum desiccator. In the clamping mechanism, the top and bottom glass plates are first brought close together to ensure that they are parallel to each other. Next, the middle metal plate is lowered to bring down the SU-8/glass, and a calculated volume (based on the final thickness) of PDMS mixture is transferred onto the mold and carefully spread to cover all the SU-8 patterns with a cleanroom swab (Texwipe Co LLC), followed by a second degassing step to remove air bubbles. The stage is then raised slowly until the gap distance between two glass plates is equal to the desired final thickness of the PDMS membrane (by referring to the stage micrometer), and the whole assembly is baked at 60 °C for 4 h in a convection oven (DX-302C, Yamato Scientific America Inc.). A relatively low curing temperature is chosen to minimize the bulging of the four-edge-clamped top glass plate due to thermal expansion as well as the shrinkage of PDMS after curing. After curing, the glass plates sandwiching the cured PDMS are released from the mechanism and are separated from each other by slowly prying with a razor blade (VWR International Inc.) from one corner to create a small gap while spraying isopropanol (IPA) into the gap. After separation, the PDMS can be slowly peeled off from the SU-8/glass with the aid of sprayed IPA, cut into four 36.2 × 36.2 mm^2^ sheets (the same size as the PZT sound source) and trimmed so that the top soldering pad on the PZT (Fig. 2a) will not be covered by the PDMS membrane after bonding. The thickness of the casted PDMS membrane is measured (Fig. 3l) with a step profilometer (DektaXT, Bruker Corp.).

After that, the PDMS membrane is aligned and bonded to the PZT substrate using a thin layer of SU-8 as an adhesive (Fig. 3f–h). A 3.5-μm-thick SU-8 layer is spin-coated (process B in Table 3) on the PZT and soft-baked (Fig. 3f). During spin-coating, the top soldering pad on the PZT is protected by a small piece of Kapton tape, which is peeled off after soft-baking to expose the soldering pad. The PDMS membrane is cleaned with IPA and deionized (DI) water, blow-dried with N_2_, cleaned again with Scotch Magic tape (3 M company) to remove any remaining particles, and treated with O_2_ plasma for 1 min (35 W, 265 mTorr) to ensure good adhesion to SU-8. Within 10 min after the plasma treatment, the PDMS membrane is aligned and attached to the PZT substrate under a stereomicroscope (Fig. 3g) with the aid of nickel alignment markers (Fig. S7). To tolerate potential error during manual alignment, the edge of the circular electrodes is designed to sit in the middle of the outermost air-cavity ring, whose width is 400 μm, allowing an alignment error of ±200 μm. During the process, the PDMS membrane is held above the PZT sheet with two pairs of plastic tweezers. After alignment, a part of the PDMS membrane is lowered and placed in contact with SU-8 on PZT, followed by gradually lowering the rest of the PDMS to increase the contact area. At this stage, the soft-baked SU-8 is solidified but not crosslinked; thus, if at any stage of the process, the alignment is not satisfactory or if any visible air bubbles are present, the PDMS membrane can be lifted for up to three attempts of realignment and reattachment. After three attempts, if any of them has not resulted in satisfactory alignment and attachment, the PDMS surface can be recleaned and reactivated by plasma treatment before another attempt(s) of alignment and attachment.

When the PDMS is attached to the PZT substrate, the chip is soft-baked again (from 40 to 80 °C with a ramp rate of 9 °C/min, baked for 3 min, and cooled to room temperature) during which the PDMS membrane is gently pressed against the PZT substrate, while the SU-8 liquefies and gets in good contact with the PDMS membrane as well as the rough PZT surface (with a thickness variation of ~±1 μm). After blanket exposure and post-exposure bake (PEB) to crosslink SU-8 (process B in Table 3), a firm bond is formed (Fig. 3h). After bonding, the PZT substrate is diced into four individual SFATs (Fig. 2a), and wires are soldered onto the soldering pads of their top and bottom electrodes, followed by Parylene deposition for electrical encapsulation (Fig. 3i). During the final Parylene deposition, there is a large pressure difference between the sealed air cavities (at atmospheric pressure of 760 Torr) and the deposition chamber (whose pressure is less than 20 mTorr), but the bonding strength is strong enough to withstand such a pressure difference.

### Microfabrication of SU-8/PDMS ACFAL

For SFAT with SU-8/PDMS ACFAL, we first cast flat PDMS using the same clamping mechanism with two blank glass plates (Fig. 4a, b). Then, 35-μm-thick SU-8 is patterned on the PZT (Fig. 4e, process C in Table 3) to define the space of the air cavities and support pillars, followed by dicing the PZT substrate into four individual chips. Next, the flat PDMS membrane is trimmed, cleaned, aligned, and attached to SU-8 on the PZT substrate under a stereomicroscope (Fig. 4f). The crosslinked SU-8 and PDMS have good cohesion. Consequently, once the PDMS is in contact with SU-8, it can stay firmly in place, while in the case of misalignment or air bubbles, it can also be peeled off damage-free for realignment. After wires are soldered, the SFAT is encapsulated by a Parylene deposition, which will permanently seal the PDMS and SU-8 together while maintaining close contact between the two materials (Fig. 4g). Since the air cavities are under atmospheric pressure while the deposition is carried out under vacuum, eight 35-μm-wide venting channels (whose total area is negligibly small) are created on the bottom SU-8 layer (Fig. 2b, h) for pressure equalization during the Parylene deposition. After deposition, the air cavities will be sealed at a close-to-vacuum pressure, and thus, the higher pressure outside the lens in the ambient environment will help to press down the PDMS membrane against SU-8, ensuring close contact, while the support pillars and the more rigid Parylene layer prevent the air cavities from collapsing. During our tests, no delamination between the layers or collapsing of air cavities was noticed.

### Microfabrication of SU-8 ACFAL

Based on a previously reported SU-8 bonding method^32^, we first attach a polyester (PET) film with adhesive on one side of a 4-inch square glass plate (Fig. 5a). The adhesive-backed PET film is from a double-sided thermal release tape with PET liners on both sides (Revealpha 3195 M, Semiconductor Equipment Corp.), whose release temperature (120 °C) is much higher than the maximal baking temperature of 90 °C, and thus functions as a normal tape during processing (although we choose this tape because it is readily available in our lab, other PET tapes with nonstick surface treatment should also work). The PET liners (which can be easily separated from the adhesive after processing) on the two sides have different thicknesses of 38 and 75 μm, which are used to support the top SU-8 layers of 30 μm (for P284) and 10 μm (for P45) during fabrication (with the other side of the PET liner being removed), respectively. Then, SU-8 is spin-coated on the PET film and soft-baked with a long baking time to prevent the filling of the air cavities due to gravity during the later bonding process (Fig. 5a, process D and process F in Table 2 for 10-μm-thick and 30-μm-thick SU-8, respectively). After that, the SU-8-coated PET film is carefully separated from the adhesive layer (Fig. 5b), and the film is cut and trimmed to fit the PZT substrate. In one batch, films for 16 lenses (covering four PZT substrates) can be made.

On the PZT substrate, a bottom layer of SU-8 is created through photolithography (Fig. 5f, process C and process E in Table 3 for 35-μm-thick and 250-μm-thick SU-8, respectively). Before producing the 250-μm-thick SU-8, a 3.5-μm-thick flat SU-8 adhesion-promotion layer is deposited to prevent delamination due to the built-in tensile stress in the thick SU-8 layer (process B in Table 3). For the 250-μm-thick SU-8, after spin-coating, a thickness planarization step^33^ is carried out (not necessary for the 35-μm-thick SU-8 as its thickness is uniform enough) by spraying the edge bead removal solution (EBR-PG, Kayaku Advanced Materials) onto the SU-8 with a compact aerosol-based sprayer (Preval Inc.) from 15 cm away for 10 sec while slowly rotating the chip to ensure uniform coverage. With the addition of the EBR solution, the viscosity of SU-8 is greatly reduced, and the reduced viscosity aids the reflow of SU-8 and eliminates air bubbles, flattening the SU-8 layer as the SU-8-coated PZT substrate is rested on a leveled surface for 10 h at room temperature followed by 2 h at 40 °C while being covered by a Petri dish cover with a 1-mm-diameter hole in the center to let the EBR solution evaporate. After that, SU-8 with uniform thickness is processed according to process E in Table 3. The thickness of the SU-8 layer (Fig. 5k) is measured with a step profilometer (DektaXT, Bruker Corp.).

Next, the top and bottom SU-8 are bonded by first treating the bottom SU-8 with O_2_ plasma (35 W, 265 mTorr) for 1 min to ensure good adhesion between the two layers. Then, the thin-SU-8-coated PET film with the SU-8 side facing down is placed on top of the PZT substrate with the thick SU-8 layer facing up, and both SU-8 layers are bonded in a laminator (TCC6000, Tamerica Products Inc.) at 80 °C with a speed setting of 3. During the lamination process, the uncrosslinked top SU-8 melts and adheres to the bottom SU-8 (Fig. 5g). The use of the laminator and the flexibility of the PET film also ensure uniform bonding across the whole PZT substrate despite the thickness variation on the bottom SU-8. Then, the areas where two layers of SU-8 need to be bonded are exposed to UV light followed by a PEB step (Fig. 5h, process D and process F in Table 3 for 10-μm-thick and 30-μm-thick SU-8, respectively) to crosslink the top SU-8 film and form a strong bond. After that, the PET film could be carefully peeled off with a spray of IPA, without any damage to the bonded SU-8 layers, followed by removing the uncrosslinked SU-8 by development (Fig. 5i). After that, the PZT substrate is diced into four chips, electric wires are soldered, and the chips are sealed with Parylene for electrical encapsulation (Fig. 5j).

### Calculation of acoustic transmittance through ACFAL

The simulation of the transmittance through the non-air-cavity areas of an ACFAL is based on a 1D multilayer acoustic transmission line model^34,35^. In the model, the acoustic waves are transmitted from the PZT having an acoustic impedance of *Z*_*0*_, pass through the ACFAL having *N* layers, and reach the medium (water) having an acoustic impedance of *Z*_*N* *+* *1*_. Assuming normal incidence, each layer *i* (*i* = *1,* *2,* *…, N*, where the 1^st^ layer is the one right above the PZT and the *N*^th^ layer is the one right beneath water) within the ACFAL can be modeled as a transfer matrix described below^34^:

1 $$M_i = \left[ {\begin{array}{*{20}{c}} {\cos \left( {k_id_i} \right)} & {jZ_i\sin \left( {k_id_i} \right)} \\ {j\sin \left( {k_id_i} \right)/Z_i} & {\cos \left( {k_id_i} \right)} \end{array}} \right],i = 1,2, \ldots ,N,$$

where *j* is the unit imaginary number; *d* is the layer thickness; *Z* is the acoustic impedance; and *k* is the complex wavenumber considering the acoustic attenuation, which is expressed as:

2 $$k_i = \frac{{2\pi f}}{{c_i}} - j\alpha _i,$$

where *f* is the frequency (2.32 MHz); *c* is the sound velocity; and α is the acoustic attenuation coefficient in Np/m. The properties of the materials used in the calculation are listed in Table S1.

Through derivation of acoustic transmission line equations^34^, the acoustic pressure *P* and acoustic velocity *V* in PZT and water can be correlated with the following equation:

3 $$\left[ {\begin{array}{*{20}{c}} {P_0} \\ {V_0} \end{array}} \right] = M_1 \cdot M_2 \cdot \ldots \cdot M_N\left[ {\begin{array}{*{20}{c}} {P_{N + 1}} \\ {V_{N + 1}} \end{array}} \right],$$

Let

4 $$M = \left[ {\begin{array}{*{20}{c}} {m_{11}} & {m_{12}} \\ {m_{21}} & {m_{22}} \end{array}} \right] = M_1 \cdot M_2 \cdot \ldots \cdot M_N.$$

Then, the acoustic transmittance *T* can be expressed as^35^:

5 $$T = \frac{{4\frac{1}{{Z_0}}\frac{1}{{Z_{N + 1}}}}}{{\left| {m_{11}\frac{1}{{Z_0}} + m_{12}\frac{1}{{Z_0}}\frac{1}{{Z_{N + 1}}} + m_{21} + m_{22}\frac{1}{{Z_{N + 1}}}} \right|^2}}$$

### Measurement of acoustic pressure and power transfer efficiency (PTE)

To measure the acoustic pressure from the SFATs, a capsule-type hydrophone (HGL-0085, Onda Corp.) is used. During measurement, immersed in a water tank, the downward-facing hydrophone is held by optical post clamps on optical posts (Newport Corp.) fixed on a five-axis high-precision movable stage consisting of two manual goniometric stages (GON-65L and GON-65U, Newport Corp.) and a three-axis motorized stage (OSMS26-XYZ, OptoSigma Corp.) The hydrophone is first scanned and aligned to the focal point of the SFAT under test, which is placed facing up below the hydrophone. The hydrophone is then scanned along the central vertical axis to determine the position of the focal plane (Fig. 6c) and along the central lateral axis at the focal plane (Fig. 6d) to measure the lateral acoustic pressure distribution. During the hydrophone measurement, a function generator (AFG-3252, Tektronix Inc.) is used to produce pulsed sinusoidal voltage signals at the measured anti-resonant frequency of each device with six cycles of sinusoidal waves per pulse, which are amplified by a power amplifier (75A250, Amplifier Research Corp.) and applied to the SFAT. During measurement, an oscilloscope (MDO3014, Tektronix Inc.) is used to simultaneously monitor the applied voltage after a 10:1 voltage attenuator (TA197, Pico Technology LLC), as well as the signal from the hydrophone after a 20-dB preamplifier (AH-2010, Onda Corp.). For each SFAT under test, the input voltage level from the function generator is adjusted so that the applied voltage on the SFAT is measured to be 40 V_pp_.

To calculate the PTE, we first estimate the total acoustic power. For that, the measured acoustic pressure *P* is converted to the intensity *I* using the equation below^31^:

6 $$I = \frac{{P^2}}{{2Z_{{\mathrm{ac}}}}}$$

where *Z*_ac_ is the acoustic impedance of water (1.48 MRayl). Since most power is concentrated in the focal zone, assuming that the pressure distribution is axisymmetric along the central vertical axis, the estimated acoustic power is calculated through a surface integral near the focal point over the focal plane, with a radial distance from the central axis ranging from 0 to 1.1 mm. The intensity at a certain radial distance is estimated by taking the average of the measured intensity values on the left and right sides of the 1D intensity distribution, which is calculated from Fig. 6d using Eq. (6). Thus, the power can be calculated through the following equation:

7 $${\mathrm{Output}}\,{\mathrm{acoustic}}\,{\mathrm{power}} \approx \mathop {\int }\nolimits_0^{1.1\,{\mathrm{mm}}} \frac{{\left[ {I_{{\mathrm{left}}}\left( r \right) + I_{{\mathrm{right}}}\left( r \right)} \right]}}{2} \times 2\pi r\,dr$$

while the real electric power is calculated using the equation:

8 $${\mathrm{Applied}}\,{\mathrm{real}}\,{\mathrm{electric}}\,{\mathrm{power}} = \frac{{V_{{\mathrm{rms}}}^2}}{{\left| {Z_{{\mathrm{elec}}}} \right|^2}} \times R = \left( {\frac{{40V_{{\mathrm{pp}}}}}{{2\sqrt 2 }}} \right)^2 \times \frac{R}{{\left| {Z_{{\mathrm{elec}}}} \right|^2}}$$

where *R* is the real part of the measured electric impedance *Z*_elec_. Finally, the PTE is calculated as:

9 $${\mathrm{PTE}} = \frac{{{\mathrm{Output}}\,{\mathrm{acoustic}}\,{\mathrm{power}}}}{{{\mathrm{{Applied}}\,{\mathrm{real}}\,{\mathrm{electric}}\,{\mathrm{power}}}}} \times 100\%$$

## Supplementary information

Graphical Abstract

Clean version of revised supplemental materials

## Data Availability

The data that support the findings of this study are available from the corresponding author upon reasonable request.
