# Supplementary material for: Simple sacrificial-layer-free microfabrication processes for air-cavity Fresnel acoustic lenses (ACFALs) with improved focusing performance
Source: Microsyst Nanoeng. 2022 Jul 5;8:75. doi: 10.1038/s41378-022-00407-w (PMC9256634; doi:10.1038/s41378-022-00407-w)
Supplement: Supplementary file 2 — Clean version of revised supplemental materials [file 41378_2022_407_MOESM2_ESM.docx]

**Supplementary Materials**

**Method S1: FEM simulation of normalized acoustic pressure distribution**

The FEM simulation of the acoustic pressure distribution of an ideal ACFAL (Fig. 1d and 1e) is carried out in the Pressure Acoustics module of COMSOL Multiphysics (COMSOL Inc.) at 2.32 MHz with a free triangular mesh having a maximum element size of 60 μm (slightly less than 1/10 of the wavelength in water). For simplicity, only the water above the transducer is considered, and the acoustic waves passing through the ACFAL are modeled with normal displacement boundary conditions defined on the six non-air-cavity Fresnel circle and rings. The material properties of water used in the simulation are shown in Table S1. To save computation time and memory, two-dimensional (2D) axial symmetry is defined, where only a half of the volume cross-section is modeled, and the plots in Fig. 1d and 1e are generated through mirroring the simulated data along the central vertical axis after normalizing the pressure value with the maximal simulated pressure at the focal point.

**Method S2: Fabrication of bottom air cavity**

After the fabrication of an SFAT, the bottom air-cavity is added (Fig. S1). Two 1.5-mm-thick acrylic pieces (McMaster-Carr Supply Company) are laser-machined with a 60 W laser cutter (LG-500, Jamieson Laser LLC). The bottom acrylic piece has a circular opening in the center that is slightly larger than the circular electrode which defines the active area of the transducer, while the top piece (without any opening) seals the opening to create the air cavity. The two pieces are bonded together with a waterproof superglue (Loctite 401, Hankel Adhesives), then aligned and glued to the backside of the SFAT with the same glue.

**Method S3: Measurement of electrical impedance**

The electrical impedances of the SFATs are measured with a vector network analyzer (8753D, Hewlett Packard Inc.) through measuring one-port reflection coefficient S_11_, when the SFAT is immersed in DI water. Then the electrical impedance *Z_elec_* is calculated from the measured S_11_ (complex number) using the equation below^1^:

|  | $Z_{elec}=50 \Omega\times\frac{1+S_{11}}{1-S_{11}}$*.* | (1) |
| --- | --- | --- |

**Table S1 Material properties used in simulations and calculations.**

| **Material** | **Mass density (kg/m^3^)** | **Sound velocity (m/s)** | **Acoustic impedance (MRayl)*** | **Attenuation coefficient (Np/m @ 2.32 MHz)** |
| --- | --- | --- | --- | --- |
| Water | 1,000^2^ | 1,480^2^ | 1.48 | 0.13^2^ |
| Parylene D | 1,418^3^ | 2,135^4^ | 3.03 | 149.58^4^ |
| PDMS (Sylgard 184 10:1 mixing ratio) | 1,030^5^ | 1,076.5^6^ | 1.06 | 74.80^5^ |
| SU-8 | 1,190^7^ | 2,860^7^ | 3.40 | 133.55^8^ |
| Nickel electrode | 8,800^9^ | 5,630^9^ | 49.54 | NA** |
| PZT | 7,800^10^ | 4,640^10^ | 36.19 | NA |

* Calculated by multiplying mass density by sound velocity.

** Ignored in simulation due to the small thickness (100 nm) of nickel.

**Table S2 Comparison of the demonstrated SFATs in this work with other high-efficiency ultrasonic transducers having similar working frequencies.**

| **Referred work** | **Center frequency (MHz)** | **Device type** | **Microfa-bricated？** | **Substrate material** | **Matching/backing layer material** | **Transmission medium** | **Max power transfer efficiency (%)** | **Output pressure per 40 V_pp_ input (MPa)** |
| --- | --- | --- | --- | --- | --- | --- | --- | --- |
| Shigeta *et al.*^11^ | 1.2 | Single-element, flat | No | Not available (NA) | NA/Air | NA | 50.4* | NA (unfocused) |
| Leadbetter *et al.*^12^ | 1.07 | Single-element, flat | No | PMN-PT/EpoTek 301 composite | EpoTek 301/Air | Water | 45.0* | NA (unfocused) |
| Ozeri *et al.*^13^ | 0.65 | Single-element, flat, with multiple electrode rings driven by Gaussian-distributed excitation voltages | No | PZT-4 | Cyanoacrylate and Graphite/Air | Pig muscle tissue | 39.1* | 0.24 (40 V_pp_ applied on the innermost ring) |
| Lee *et al.*^14^ | 0.86 | Single-element, flat | No | PZT-5A | NA/Air | Water | 31.0** | 0.18 (unfocused) |
| Suzuki *et al.*^15^ | 1.0 | Single-element, flat | No | PZT | NA/NA | Water | 20.0* | NA (unfocused) |
| Kim *et al.*^16^ | 3.6 | Single-element, focused with curved surface | No | Epoxy/PMN-PT composite | NA/NA | Water/Chicken breast | 2.7* | 1.04 |
| Mazzilli *et al.*^17^ | 1.0 | 64-element spherical linear phased array | No | NA | None/Air | Tissue phantom | 1.6* | 1.38 (with 64 elements activated) |
| Wang *et al.*^18^ | 1.1 | 32-element linear phased array | No | PZT-4 | None/Air | Castor oil | 1.0** | 0.35 (square-wave drive) |
| This work | 2.3 | Single-element, flat, focused with planar air-cavity Fresnel acoustic lens | Yes | PZT-5A | Matching: PDMS, or SU-8, or SU-8/PDMS  Backing: Air | Water | 15.2 to  30.13** | 0.68 to 1.10 |

* Defined for a transmitting-receiving two-transducer system, as the ratio between the output electrical power converted by a receiving acoustic transducer and the input electrical power applied to a spatially aligned transmitting transducer.

** Defined for a single transmitting transducer, defined as the ratio between the output acoustic power and the applied electric power.


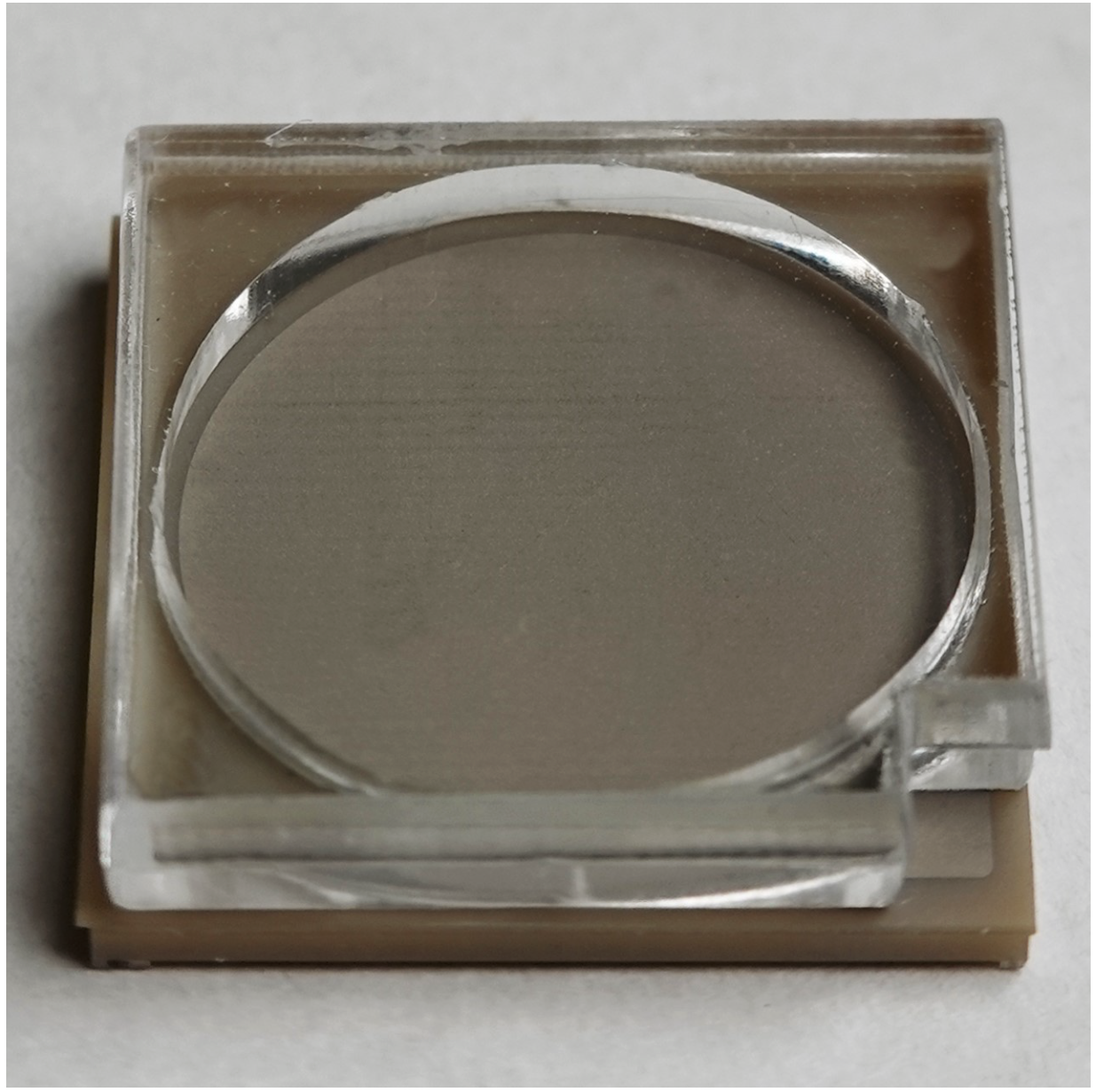


**Fig. S1 Photo of an air-cavity formed with laser-machined acrylic sheets covering the circular active area at the back of a 16-mm-side-length SFAT.**


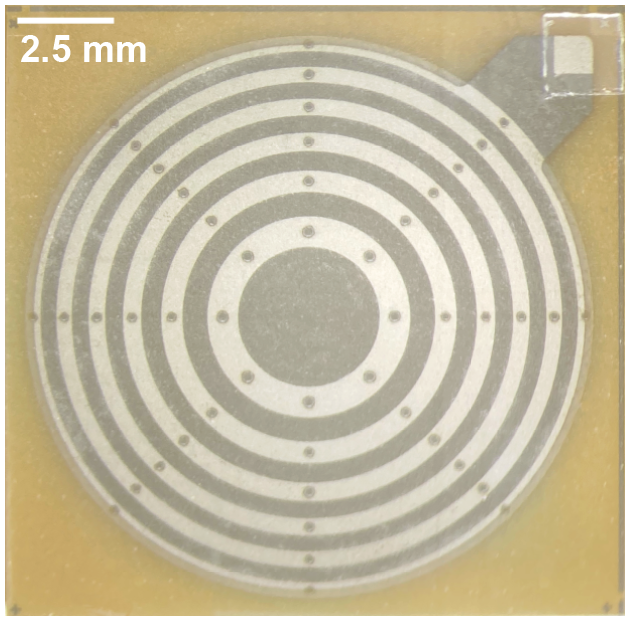


**Fig. S2 Top-view photo of an SFAT with a Parylene ACFAL before electric wires are soldered.**


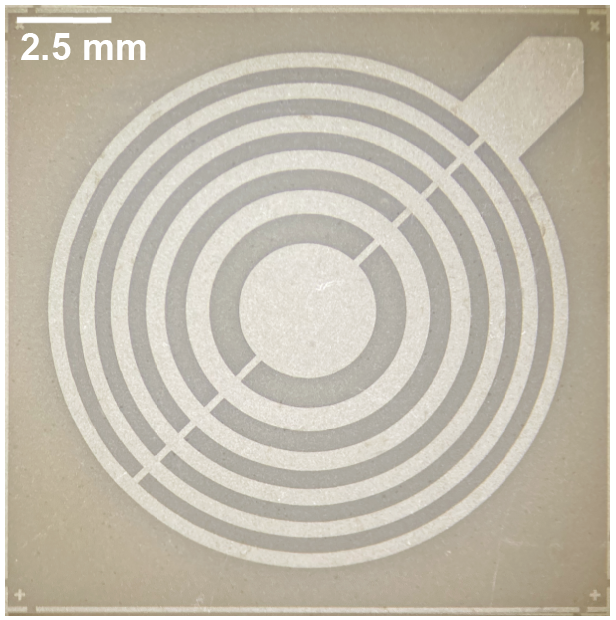


**Fig. S3 Top-view photo of an SFAT based on patterned electrode rings before electric wires are soldered.**


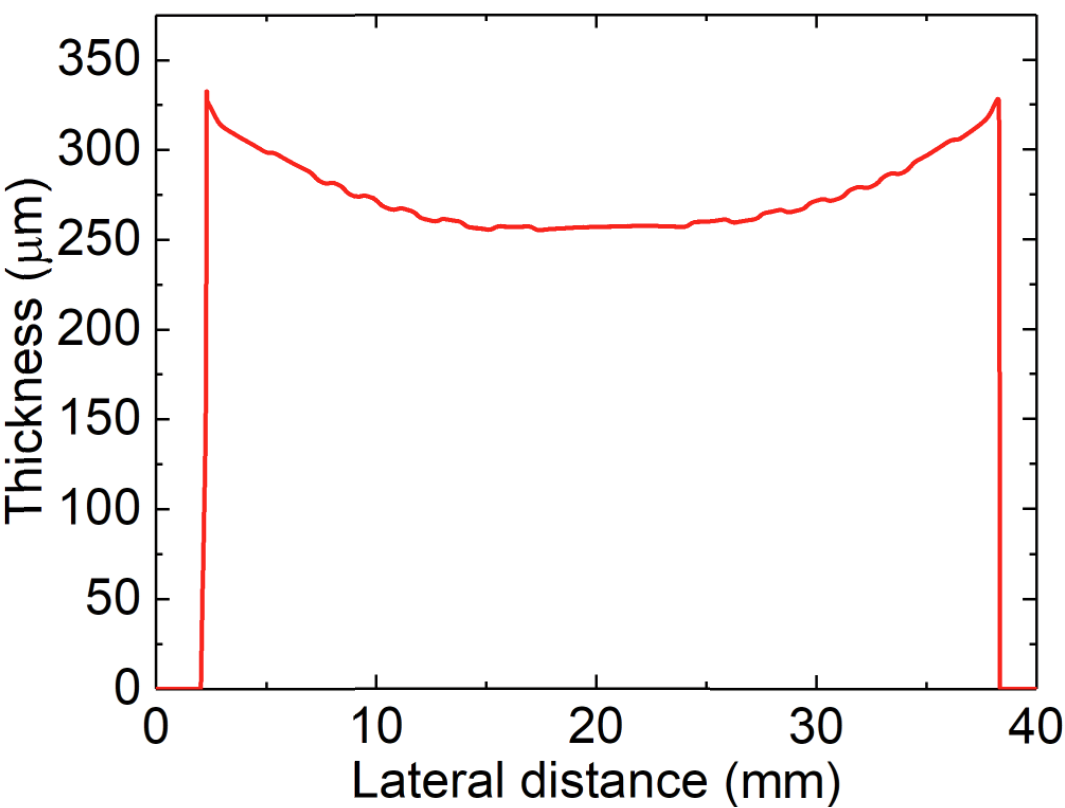


**Fig. S4 Measured thickness profile of a PDMS membrane created from spin-coating on an SU-8/glass mold, showing poor** **thickness uniformity due to the formation of edge bead.**


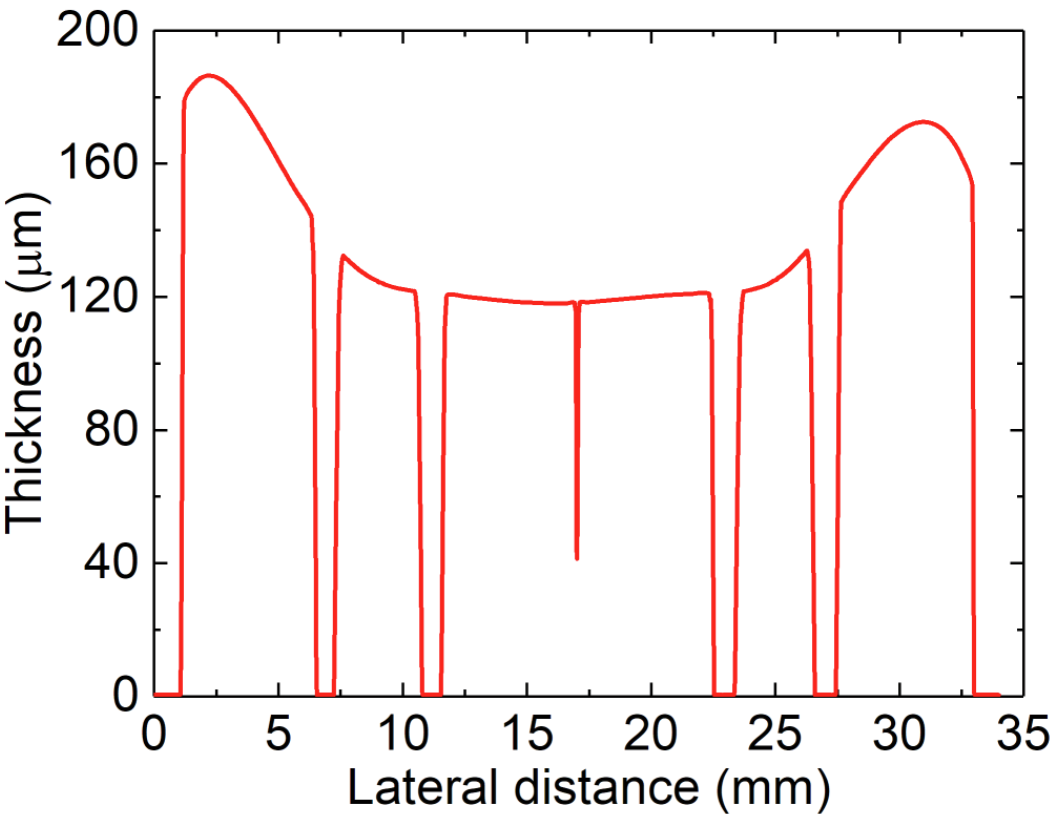


**Fig. S5 Measured thickness profile of an SU-8 layer formed without the planarization step, showing poor thickness uniformity due to the formation of edge beads, especially when the viscosity of the SU-8 is high.**


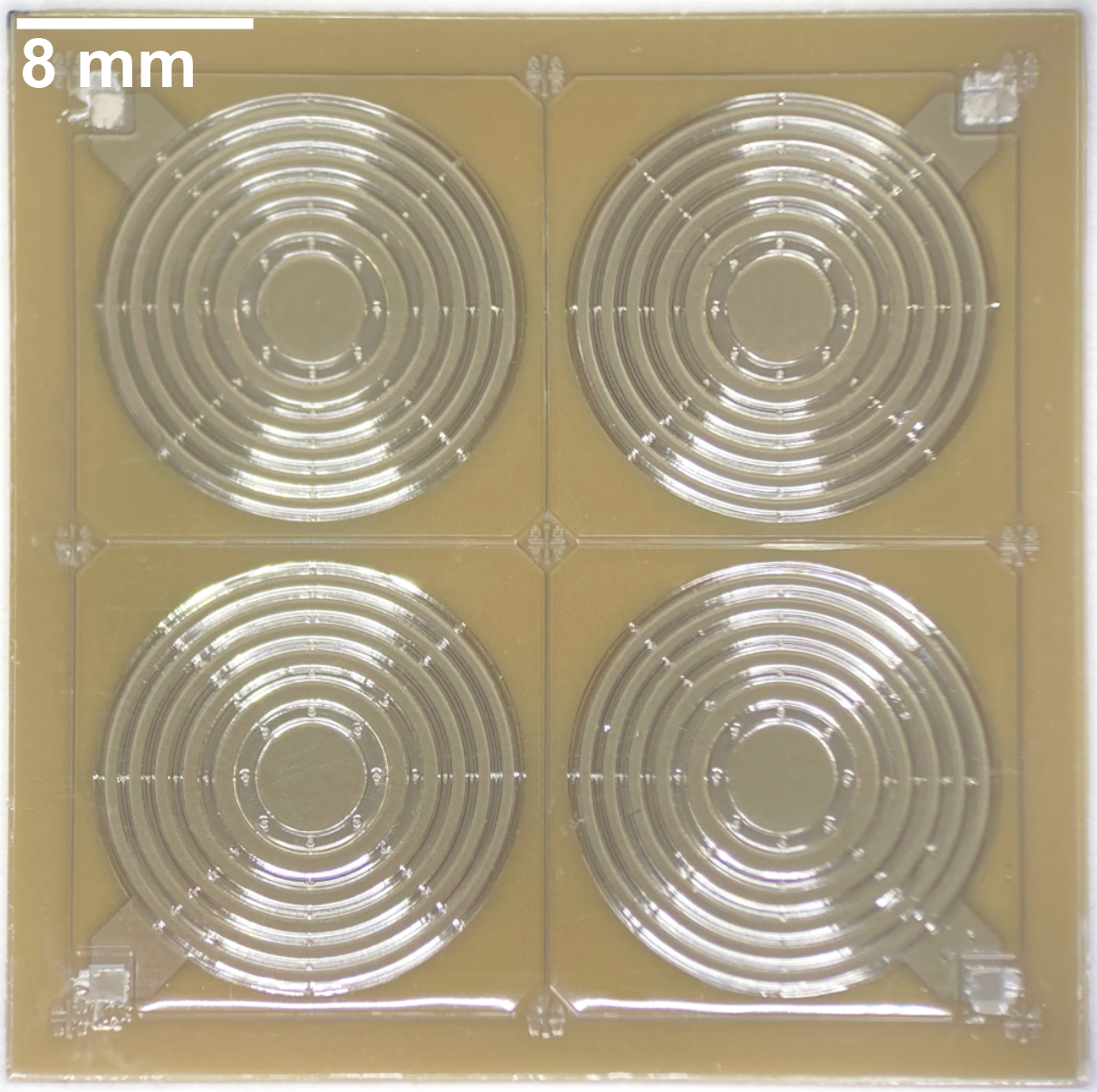


**Fig. S6 Top-view photo showing four fabricated SU-8-ACFAL-based SFATs with 16 mm side length on one square PZT sheet with 36.2 mm side length before dicing.**


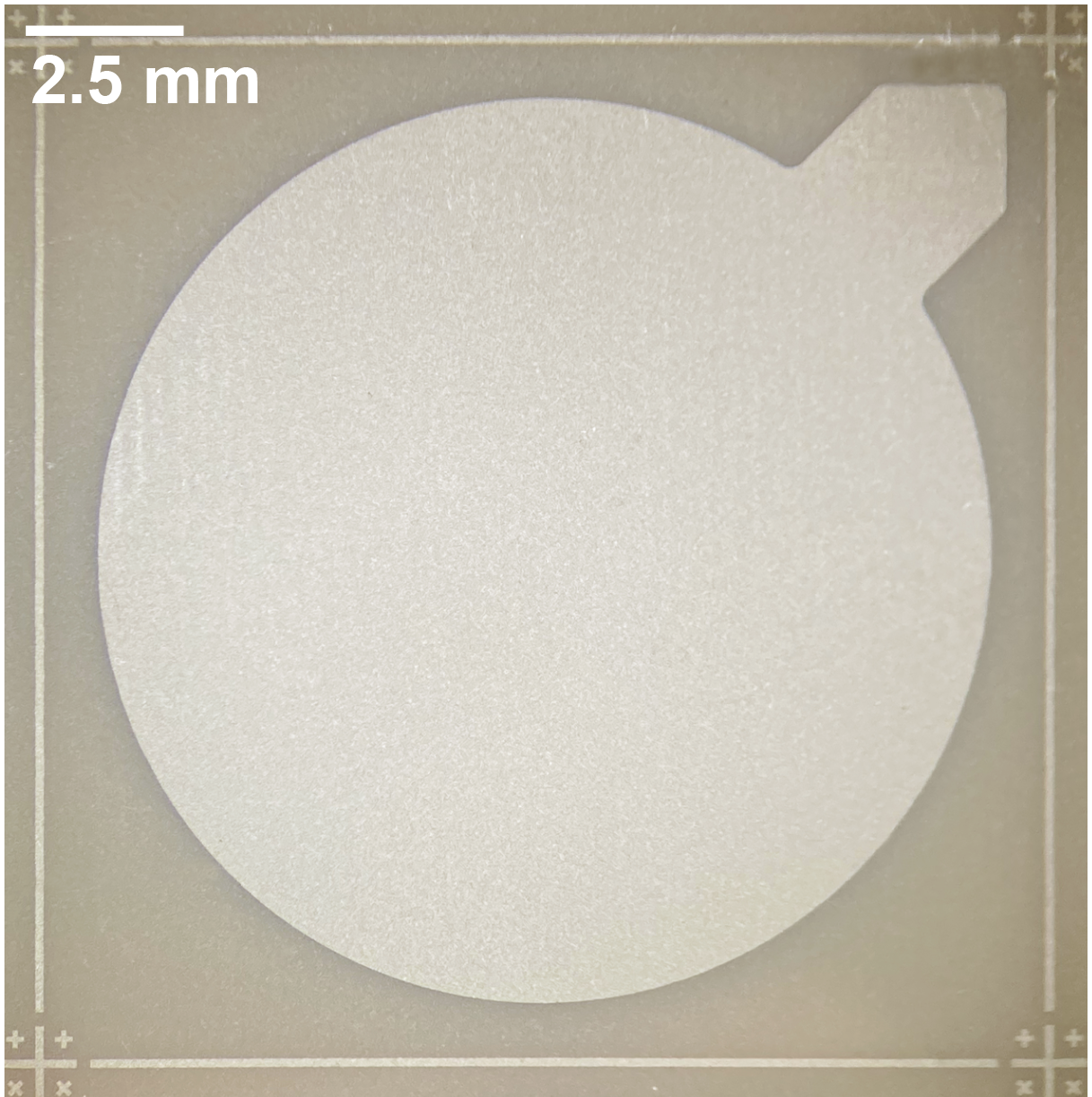


**Fig. S7 Top-view photo showing the top electrode, the soldering pad, and alignment markers on a PZT sound source.** The same electrode pattern is used in all ACFAL-based SFATs described in this paper.

**Supplemental References**

1. Maxim Integrated Products, Inc. Converting s-parameters from 50 Ω to 75 Ω impedance. https://www.maximintegrated.com/en/design/technical-documents/tutorials/2/2866.html.

2. Shung, K. K. *Diagnostic ultrasound: Imaging and blood flow measurements*. (CRC Press, 2005).

3. Nguyen, N.-T., Wereley, S. T. & Shaegh, S. A. M. *Fundamentals and applications of microfluidics*. (Artech House, 2019).

4. Levassort, F., Tran-Huu-Hue, L.-P., Marechal, P., Ringgaard, E. & Lethiecq, M. Characterisation of thin layers of parylene at high frequency using PZT thick film resonators. *J. Eur. Ceram. Soc.* **25**, 2985–2989 (2005).

5. Xu, G. *et al.* Acoustic characterization of polydimethylsiloxane for microscale acoustofluidics. *Phys. Rev. Appl.* **13**, 054069 (2020).

6. Tsou, J. K., Liu, J., Barakat, A. I. & Insana, M. F. Role of ultrasonic shear rate estimation errors in assessing inflammatory response and vascular risk. *Ultrasound Med. Biol.* **34**, 963–972 (2008).

7. Gorishnyy, T., Jang, J.-H., Koh, C. & Thomas, E. L. Direct observation of a hypersonic band gap in two-dimensional single crystalline phononic structures. *Appl. Phys. Lett.* **91**, 121915 (2007).

8. Wang, S. *et al.* SU-8-based nanocomposites for acoustical matching layer. *IEEE Trans. Ultrason. Ferroelectr. Freq. Control* **56**, 1483–1489 (2009).

9. Krautkrämer, J. & Krautkrämer, H. *Ultrasonic Testing of Materials*. 620 (Springer-Verlag, 1983).

10. Piezo.com. Piezoelectric Material Technical Datasheet (Typical Values). https://info.piezo.com/hubfs/Data-Sheets/piezo-material-properties-data-sheet-20201112.pdf.

11. Shigeta, Y., Hori, Y., Fujimori, K., Tsuruta, K. & Nogi, S. Development of highly efficient transducer for wireless power transmission system by ultrasonic. in *2011 IEEE MTT-S International Microwave Workshop Series on Innovative Wireless Power Transmission: Technologies, Systems, and Applications* 171–174 (2011).

12. Leadbetter, J., Brown, J. A. & Adamson, R. B. The design of ultrasonic lead magnesium niobate-lead titanate (PMN-PT) composite transducers for power and signal delivery to implanted hearing aids. *Proc. Mtgs. Acoust.* **19**, 030029 (2013).

13. Ozeri, S., Shmilovitz, D., Singer, S. & Wang, C.-C. Ultrasonic transcutaneous energy transfer using a continuous wave 650 kHz Gaussian shaded transmitter. *Ultrasonics* **50**, 666–674 (2010).

14. Lee, H. J. *et al.* Piezoelectric charging and wireless communication. in *Sensors and Smart Structures Technologies for Civil, Mechanical, and Aerospace Systems 2018* vol. 10598 252–264 (SPIE, 2018).

15. Suzuki, S.-N. *et al.* Power and Interactive Information Transmission to Implanted Medical Device Using Ultrasonic. *Jpn. J. Appl. Phys.* **41**, 3600 (2002).

16. Kim, K., Jang, S. G., Lim, H. G., Kim, H. H. & Park, S.-M. Acoustic Power Transfer Using Self-Focused Transducers for Miniaturized Implantable Neurostimulators. *IEEE Access* **9**, 153850–153862 (2021).

17. Mazzilli, F., Lafon, C. & Dehollain, C. A 10.5 cm ultrasound link for deep implanted medical devices. *IEEE Trans. Biomed. Circuits Syst.* **8**, 738–750 (2014).

18. Wang, M. L. *et al.* Closed-loop ultrasonic power and communication with multiple miniaturized active implantable medical devices. in *2017 IEEE International Ultrasonics Symposium (IUS)* 1–4 (2017).
