# Supplementary material for: Simple sacrificial-layer-free microfabrication processes for air-cavity Fresnel acoustic lenses (ACFALs) with improved focusing performance
Source: Microsyst Nanoeng. 2022 Jul 5;8:75. doi: 10.1038/s41378-022-00407-w (PMC9256634; doi:10.1038/s41378-022-00407-w)

SFAT = Sound Source + ACFAL

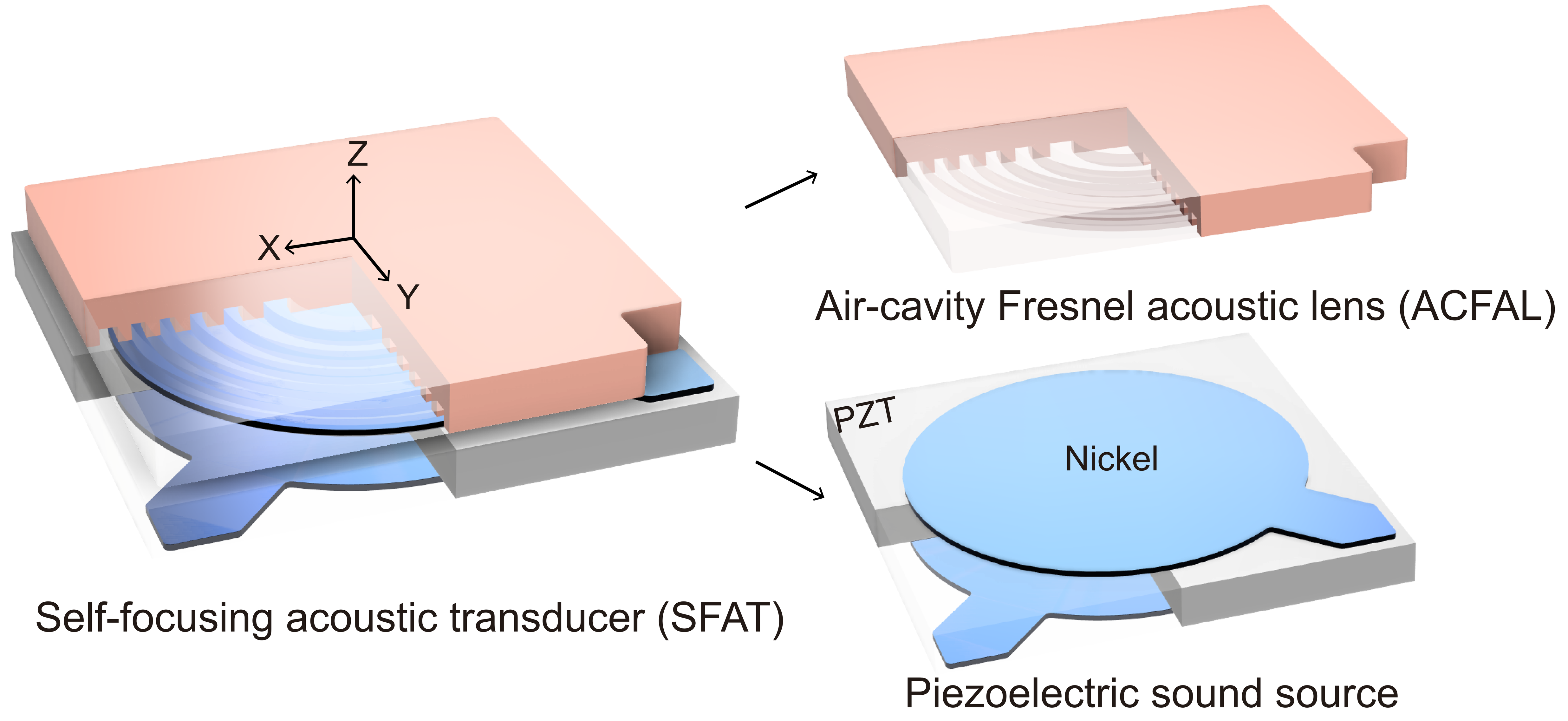

Optimization & Precise Control of Lens Thickness for Maximizing Focusing Performance

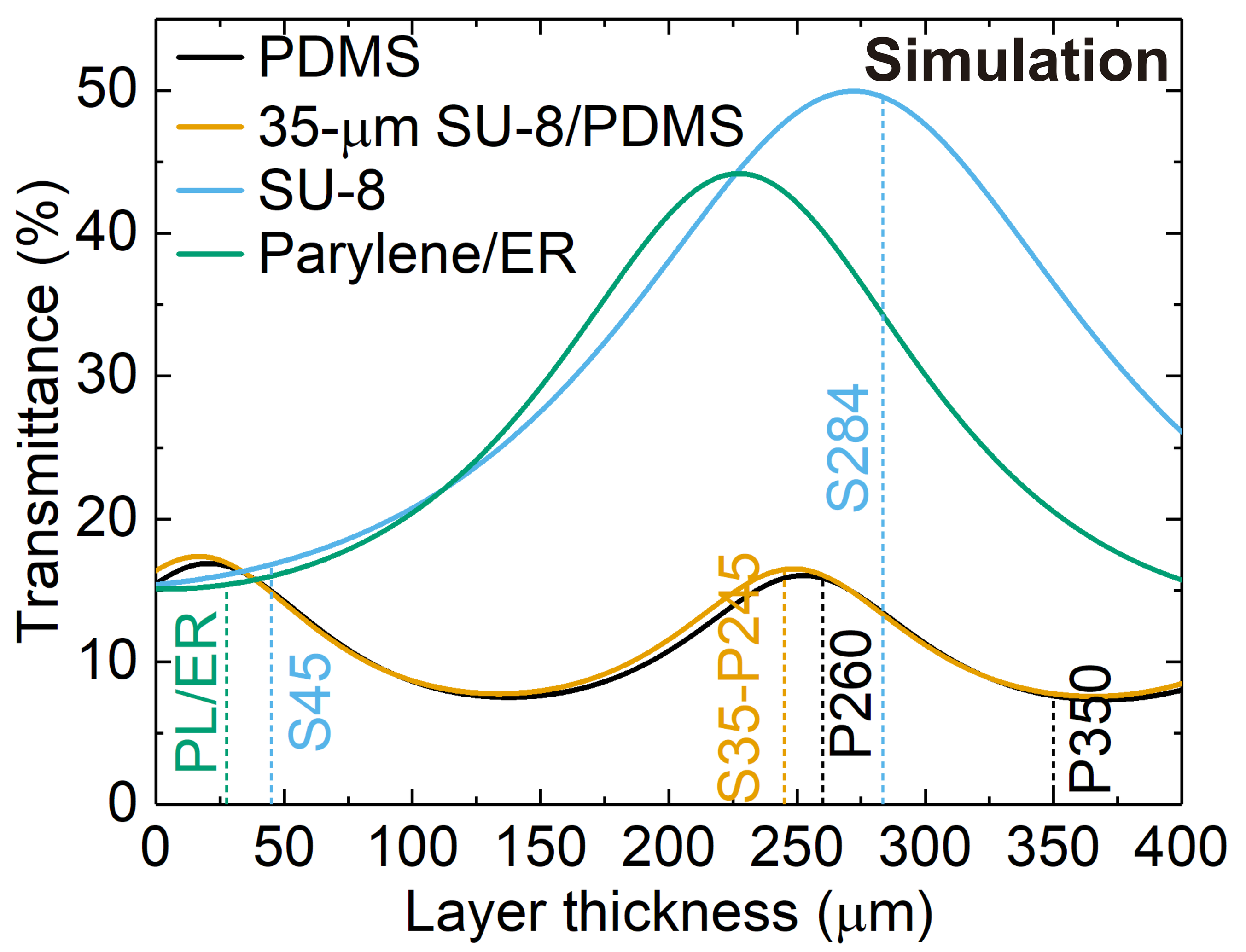

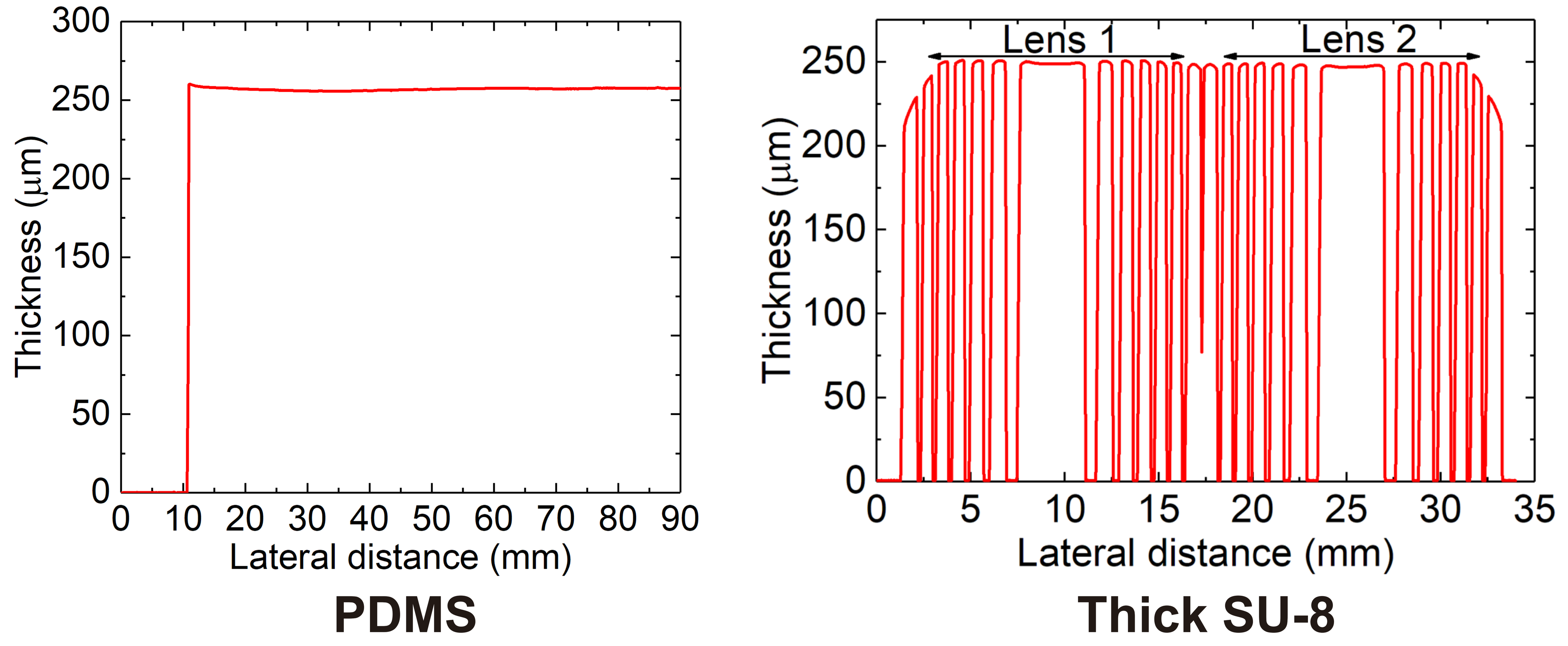

Sacrificial-Layer-Free Microfabrication: 2X–4X Faster

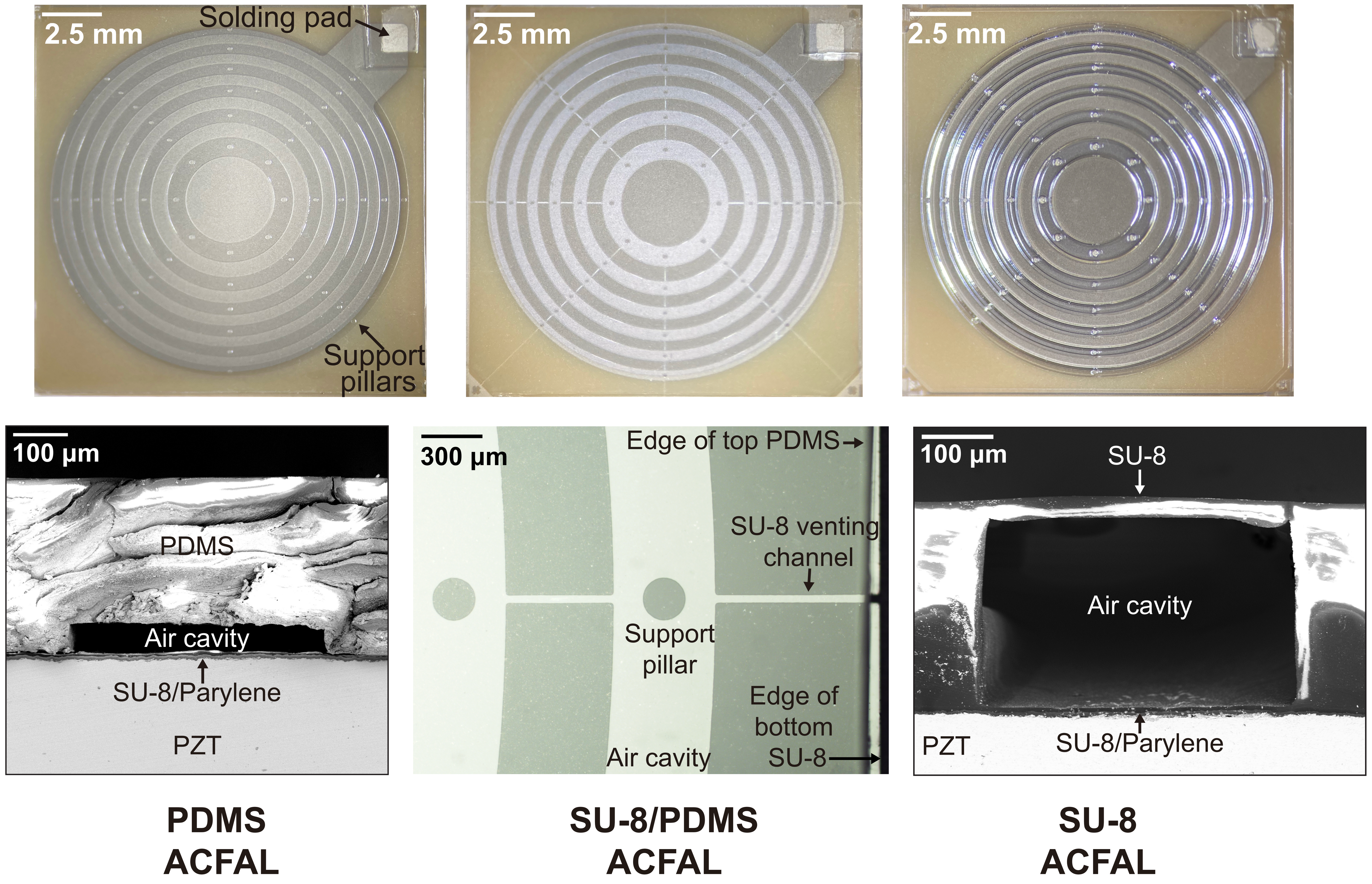

Improved Focusing Performance: Up to 2X

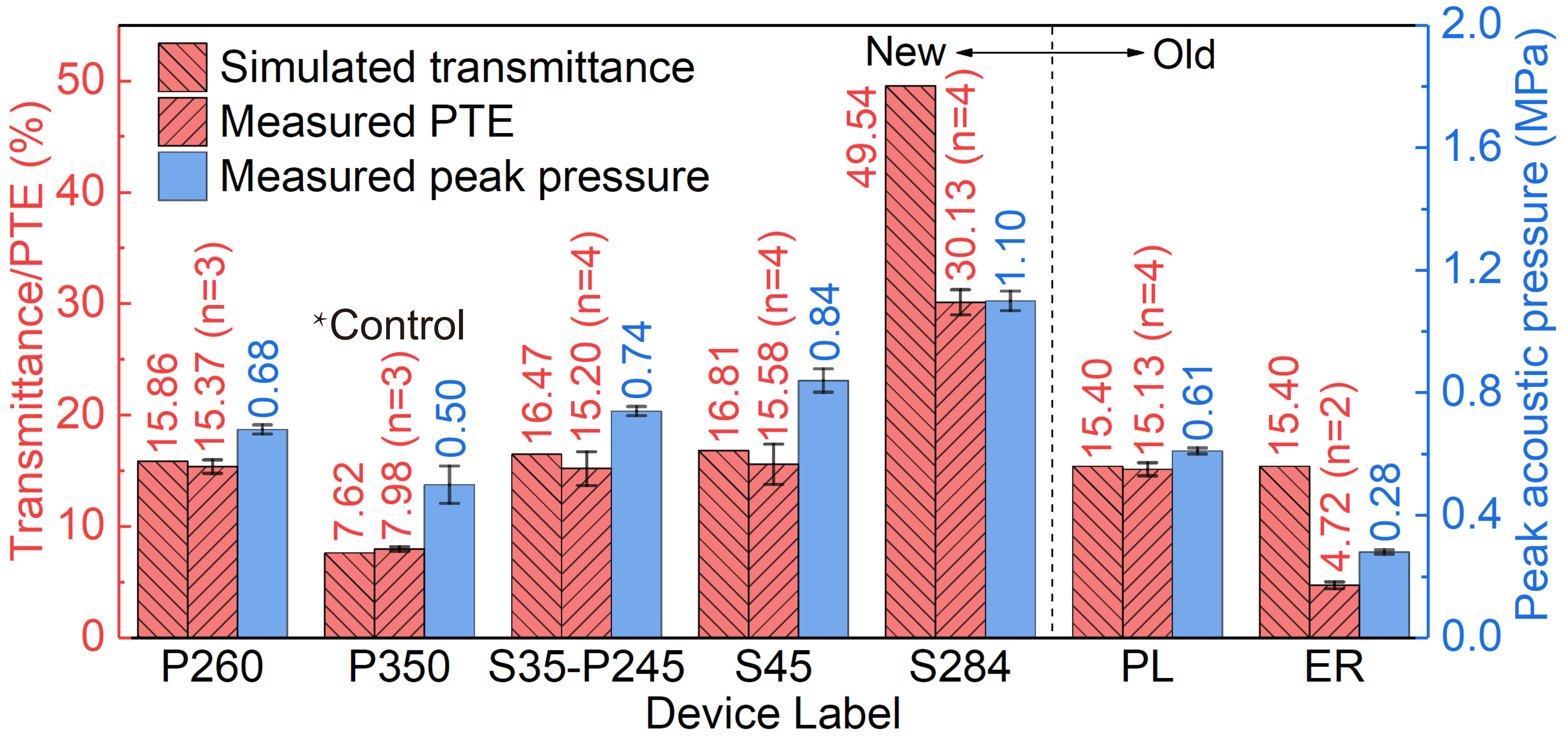

Supplement: Supplementary file 1 — Graphical Abstract [file 41378_2022_407_MOESM1_ESM.pdf]
